# Triphenylamine-Merocyanine-Based D1-A1-π-A2/A3-D2 Chromophore System: Synthesis, Optoelectronic, and Theoretical Studies

**DOI:** 10.3390/ijms20071621

**Published:** 2019-04-01

**Authors:** Pedada Srinivasa Rao, Avinash L. Puyad, Sidhanath V. Bhosale, Sheshanath V. Bhosale

**Affiliations:** 1 Polymers and Functional Materials Division, CSIR-Indian Institute of Chemical Technology, Hyderabad 500007, India; srinuchem99@gmail.com; 2 Academy of Scientific and Innovative Research (AcSIR), Ghaziabad 201002, India; 3 School of Chemical Sciences, Swami Ramanand Teerth Marathwada University, Nanded 431606, India; avinashlpuyad@gmail.com; 4 Department of Chemistry, Goa University, Taleigao Plateau, Goa 403206, India

**Keywords:** donor–acceptor, cyclic voltammograms, triphenylamine, tetracyanoethylene, 7,7,8,8-tetracyanoquinodimethane

## Abstract

donor–acceptorDonor–acceptor–π–acceptor–donor (D1-A1-π-A2/A3-D2)-type small molecules, such TPA-MC-2 and TPA-MC-3, were designed and synthesized starting from donor-substituted alkynes (TPA-MC-1) via [2 + 2] cycloaddition−retroelectrocyclization reaction with tetracyanoethylene (TCNE) and 7,7,8,8-tetracyanoquinodimethane (TCNQ) units, respectively. TPA-MC-2 and TPA-MC-3 chromophores differ on the A2/A3 acceptor subunit, which is 1,1,4,4-tetracyanobutadiene (TCBD) and a dicyanoquinodicyanomethane (DCQDCM), respectively. Both the derivative bearing same donors D1 (triphenylamine) and D2 (trimethylindolinm) and also same A1 (monocyano) as an acceptor, tetracyano with an aryl rings as the π-bridging moiety. The incorporation of TCNE and TCNQ as strong electron withdrawing units led to strong intramolecular charge-transfer (ICT) interactions, resulting in lower LUMO energy levels. Comparative UV–Vis absorption, fluorescence emission, and electrochemical and computational studies were performed to understand the effects of the TCNE and TCNQ subunits incorporated on TPA-MC-2 and TPA-MC-3, respectively.

## 1. Introduction

The fabrication of low-cost electronic devices such as bulk heterojunction (BHJ) and organic solar cells (OSCs) has become important in commercial applications because of their light-absorbing characteristics. Therefore, the band gap engineering of such chromophores aims to improve the efficiency of the devices through the manipulation of highest occupied molecular orbitals (HOMO) and lowest unoccupied molecular orbitals (LUMO), thus, has attracted wide attention of researchers [1,2]. From literature study it is clear that OSCs will lead to build promising next generation clean and renewable energy devices [3]. The optoelectronic properties of chromophore could be altered by varying the strength of donor (D) or acceptor (A) subunits [4,5,6,7,8,9,10]. To achieve this researcher employed the cross-conjugation reaction to tune the LUMO energy level of chromophores [11,12,13,14,15]. To fulfill this requirement, [2 + 2] cycloaddition–retroelectrocyclization reaction of electron withdrawing moieties such as tetracyanoethene (TCNE) and 7,7,8,8-tetracyanoquinodimethane (TCNQ) with π-conjugated system comprised of activated alkynes were used [11,16,17,18,19,20,21,22]. Nevertheless, the chromophores bearing TCBD and dicyanoquinodicyanomethane (DCQDCM) in backbone display low LUMO, which is necessary to improve the OSCs device performance because strong intramolecular charge-transfer (ICT) interaction observed in D and TCNQ and DCQDCM acceptors [11,12]. The literature search revealed that TCBD and DCQDCM incorporated D-A chromophores and polymers were employed to fabricate the nonlinear, molecular batteries, and also optoelectronic materials [10,22,23,24,25].

Merocyanine (MC) dyes are the traditional colorant exhibits high absorption coefficient, tunable absorption properties and applied as textile colorant. They are attractive dyes for high technological applications such as nonlinear optics and photorefractive materials due to their polarizabilities and dipole moments [26,27,28,29]. Merocyanine dyes are also promising candidates for dye-sensitized solar cells (DSSCs) [30,31,32,33,34,35], photorefractive applications [36,37,38], and solution-processed BHJ OSCs [39,40]. Würthner and coworkers extensively investigated bulk heterojunction (BHJ) OSCs application of merocyanine dyes [41,42,43].

On the other hand, triphenylamine (TPA) has been recognized as a very promising electron-donor unit due to its charge transport characteristics [44,45,46,47,48,49]. TPA and its derivatives were used in various fields such as electrochromism, organic electronics, two-photon absorption, DSSCs, and organic photovoltaics [50,51,52,53,54,55].

Considering the above properties of MC and TPA, herein, we report synthesis of two chromophores, TPA-MC-2 and TPA-MC-3, prepared by the Sonogashira–Hagihara coupling reaction followed by [2 + 2] cycloaddition–retroelectrocyclization click chemistry reaction [56]. The thermal, absorption, photoluminescence, electrochemical, and theoretical studies of these derivatives were investigated in detail. Furthermore, we also examined the effect of enhanced π-conjugation on these properties to find out energy band gaps.

## 2. Results and Discussion

### 2.1. Design

We designed and synthesized small molecule conjugates D1-A1-π-A2/A3-D2 composed of electron donor and acceptor moieties. Here we presume that the designed molecule D1-A1-π-A2/A3-D2 will display broad range absorption and electron accepting characteristics, thus, such a design strategy comprising alternate donor–acceptor subunits is useful to improve the photovoltaic performance and could reduce the energy band gap [57,58,59]. The designed D1-A1-π-A2-D2 systems are rarely explored, which is made up of four different subunits, i.e., the MC donor unit at one terminal end and the TPA donor subunit at another end, and both the donor moieties are covalently linked at center with two different acceptor subunits, such as –CN and either of TCBD or DCQDCM chromophore via an aromatic π-system, respectively.

### 2.2. Synthesis and Characterization

**Scheme 1:**
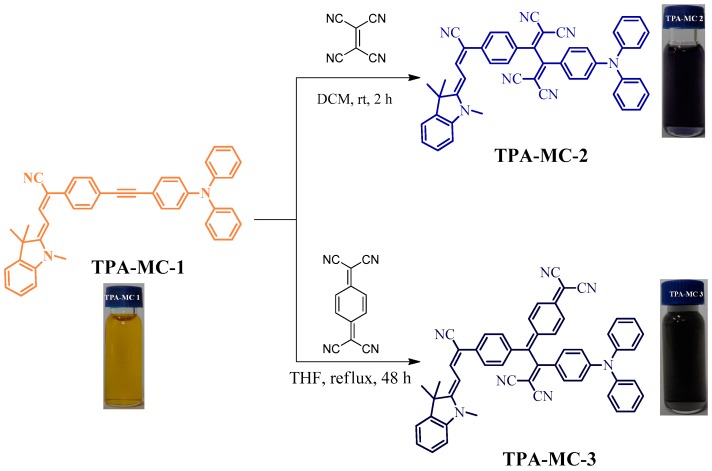
Synthesis of target donor–acceptordonor–acceptor–π–acceptor–donor systems.

**Scheme 2:**
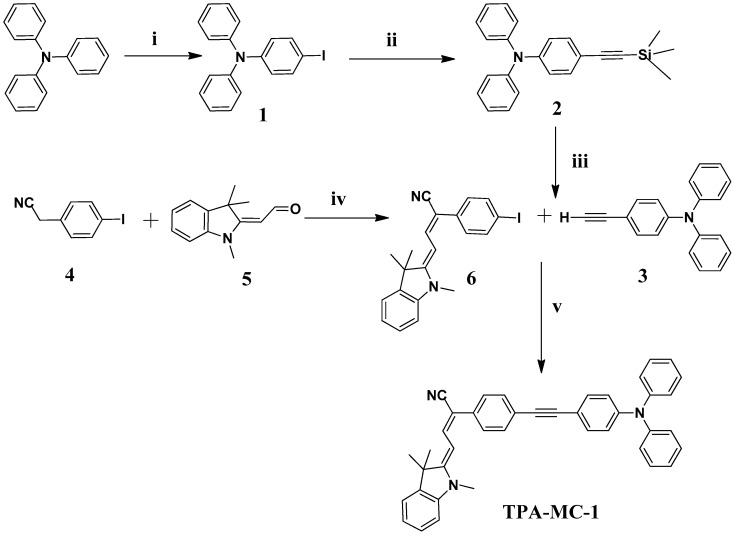
Reagents and conditions: (i) NIS, CHCl_3_+CH_3_COOH (1:1), rt, 12 h; (ii) PdCl_2_ (PPh_3_)_2_/CuI, Et_3_N, dry THF, TMSA, rt, 12 h; (iii) K_2_CO_3_, MeOH+THF (1:1), rt, 4 h; (iv) piperidine, dry THF, 12 h; and (v) Pd(PPh_3_)_4_ /CuI, DIPEA, dry THF, reflux, 12 h.

The synthesis of the designed target molecules TPA-MC-2 and TPA-MC-3 is shown in Scheme 1 and synthesis of starting material TPA-MC-1 as shown is Scheme 2. The intermediate 4-ethynyl-N,N-diphenylaniline **3** was synthesized from triphenylamine in three steps as shown in Scheme 2 [60], whereas (2E,4E)-2-(4-iodophenyl)-4-(1,3,3-trimethylindolin-2-ylidene)but-2-enenitrile **6** was prepared from 4-iodo phenyl acetonitrile **4** and 2-(1, 3, 3-trimethylindolin-2-ylidene) acetaldehyde **5** via Knoevenagel condensation reaction [61]. The Sonogashira–Hagihara coupling method was used to prepare starting TPA-MC-1 derivative from compound **3** and **6** [62,63,64]. The obtained TPA-MC-1 was further subject to react with TCNE and TCNQ through [2+2] cycloaddition–retroelectrocyclization click chemistry to yield TPA-MC-2 and TPA-MC-3, respectively (Scheme 1). The chemical structures of TPA-MC-1, TPA-MC-2, and TPA-MC-3 were confirmed by modern spectroscopic techniques, such as FT-IR, ^1^H NMR, ^13^C NMR spectroscopy, and mass and high-resolution mass spectrometry (HRMS) (Figures S8–S29).

The thermal stability of the three synthesized compounds—TPA-MC-1, TPA-MC-2 and TPA-MC-3—were determined using thermogravimetric analysis (TGA) (Figures S1–S3). The samples were heated up to 600 °C at a rate of 10 °C/min under nitrogen atmosphere. TPA-MC-1 exhibits the wt. loss of 5% at 331.88 °C (Figure S1), whereas TPA-MC-2 and TPA-MC-3 display the 5% wt. loss at 257.66 °C (Figure S2) and 185.40 °C (Figure S3), respectively. These TGA results revealed that all three compounds—TPA-MC-1, TPA-MC-2, and TPA-MC-3—are thermally stable.

### 2.3. Theoretical Calculations

**Figure 1:**
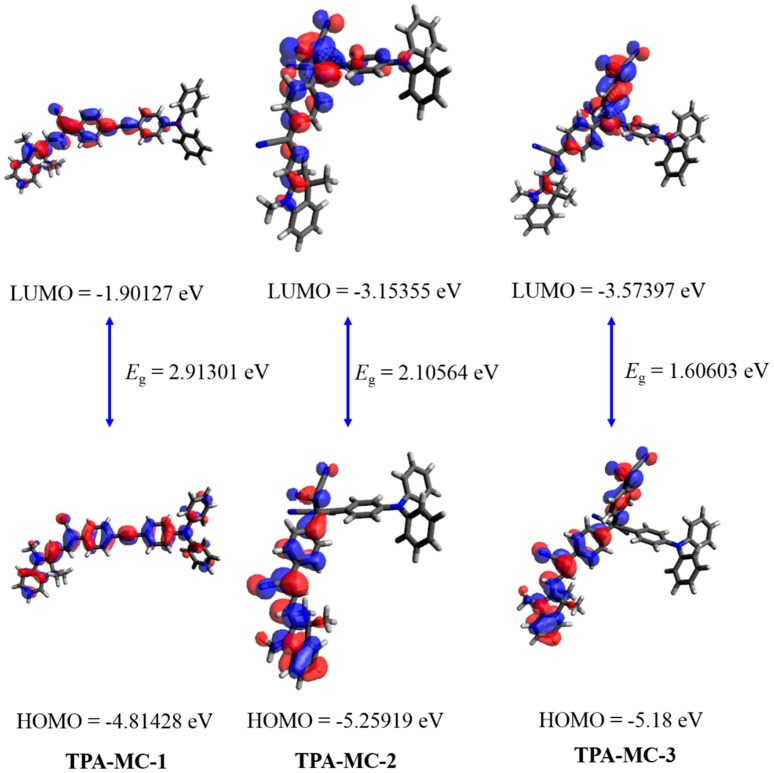
The molecular orbital diagrams of TPA-MC-1, TPA-MC-2, and TPA-MC-3.

The Gaussian 09 ab initio/DFT quantum chemical calculations were employed to examine the electronic properties of molecular structures [65]. The B3LYP/6-31G(d) level of theory and frequency calculations were carried out to optimize the geometry of TPA-MC-1, TPA-MC-2, and TPA-MC-3 molecules, respectively. The obtained geometries of TPA-MC-1, TPA-MC-2, and TPA-MC-3 via B3LYP/6-31G(d) were further investigated for the better treatment of charge-transfer excitations through time-dependent density functional theory (TD-DFT) and the results are illustrated in Table S1 [66,67]. The effect of dichloromethane was included by means of the polarizable continuum model (PCM). The TD-DFT results show that TPA-MC-1 gives an absorption band at 480 nm (Figure S5); TPA-MC-2 shows three absorption bands at 677 nm, 541 nm, and 468 nm (Figure S6); and TPA-MC-3 shows absorption bands at 853 nm, 542 nm, and 462 nm (Figure S7). The frontier molecular orbitals (FMO) calculated at B3LYP/6-31G(d) level of theory and generated by using Avogadro as shown in Figure 1 [56,67]. The calculated electronic-density distribution of the highest occupied molecular (HOMO) orbital of TPA-MC-1 is located on the entire molecule. The lowest unoccupied molecular (LUMO) orbital level of TPA-MC-1 is distributed over MC and acetylene bond. The HOMO of TPA-MC-2 distributed over the tetracyanobutadiene (TCBD) and MC subunits, whereas the LUMO is located at TCBD subunit only. The electronic distribution of the HOMO and of TPA-MC-3 is distributed on DCQDCM and MC subunits. The LUMO of TPA-MC-3 is located only on DCQDCM unit. The HOMO and LUMO of TPA-MC-2 and TPA-MC-3 are lower than that of TPA-MC-1 and are attributed to the incorporation of TCBD and DCQDCM subunits, respectively; which, in turn, resulted in a decrease of the energy band gap (*E_g_*) from 2.91301 eV of TPA-MC-1 to 2.10564 eV of TPA-MC-2 to 1.60603 eV of TPA-MC-3. Thus, the energy levels and band gaps can be tuned by incorporating electron acceptor subunit in the backbone of TPA-MC-1 (D-A-π-D).

### 2.4. Optical and Emission Properties

**Figure 2:**
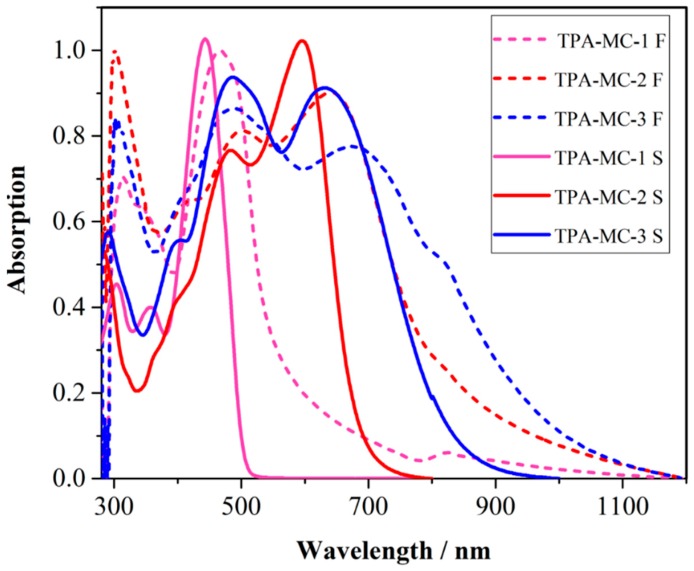
UV–Vis spectra of TPA-MC-1, TPA-MC-2, and TPA-MC-3 in solution (solid lined) and thin film (dashed lines).

The UV–Vis absorption spectra of TPA-MC-1, TPA-MC-2, and TPA-MC-3 molecules in chloroform solution as well as in thin film are depicted (Figure 2). The TPA-MC-1 molecule exhibits strong absorption spectra at 442 nm along with two more less intense peaks at 364 and 313 nm. The spectrum of TPA-MC-1 in thin film shows an absorption peaks at 445 nm and 315 nm. The peaks of thin film spectrum broadened with slight red shift. The chloroform solution of TPA-MC-2 displays a strong absorption peak at 595 nm along with two shoulder peaks at 481 nm and 282 nm, and its thin film spectrum is broadened and appeared with a bathochromic shift at 631 nm with shoulder peaks at 498 nm and 300 nm. The spectra of TPA-MC-3 show strong absorption peaks at 663 nm, 483 nm, and a less intense peak at 291 nm. The thin film spectrum of TPA-MC-3 appears at 670 nm, 485 nm, and 300 nm, indicating that the molecules pack tightly in solid-state. The absorption peaks of TPA-MC-2 and TPA-MC-3 in solution and thin films are greatly broadened as compare to TPA-MC-1, this is due to the stronger intermolecular interactions and stronger ICT effect in solid-state. The optical band gaps, *E*_g_, of TPA-MC-1, TPA-MC-2, and TPA-MC-3 were calculated from their absorption onsets 776 nm, 986 nm, and 1083 nm of the thin film, and are 1.59 eV, 1.25 eV, and 1.14 eV, respectively (Table 1). These results indicate that the incorporation of TCBD and DCQDCM chromophores at the backbone of the TPA-MC-1 displays significant influence on the optical band gaps and light harvesting ability of TPA-MC-2 and TPA-MC-3.

**Figure 3:**
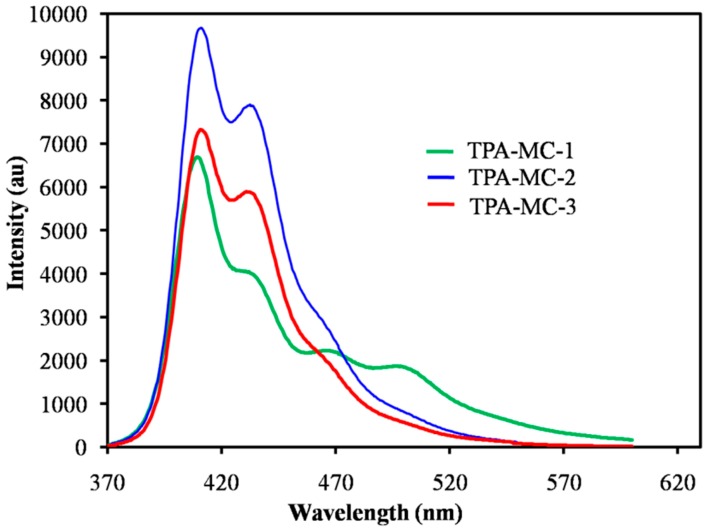
Fluorescence emission spectra of TPA-MC-1, TPA-MC-2, and TPA-MC-3 in chloroform solution (λ_ex_ = 350 nm).

We also investigated fluorescence spectroscopy of TPA-MC-1, TPA-MC-2, and TPA-MC-3 in chloroform solution upon excitation at 350 nm, as shown in Figure 3. TPA-MC-1 displays the emission main peak at 407 nm along with three smaller peaks at 434, 469, and 501 nm. The significant strong emission peaks of TPA-MC-2 appeared at 412 nm, along with two additional peaks at 434 nm and 466 nm. The solution of TPA-MC-3 showed emission peaks at 412, 433, and 468 nm, respectively. This indicates that all three molecules TPA-MC-1, TPA-MC-2, and TPA-MC-3 are light emissive. However, neither of these derivatives produced any emission in the solid thin film; this may be due to overlapping of donor–acceptor system and cause fluorescence.

### 2.5. Electrochemical Properties

**Figure 4:**
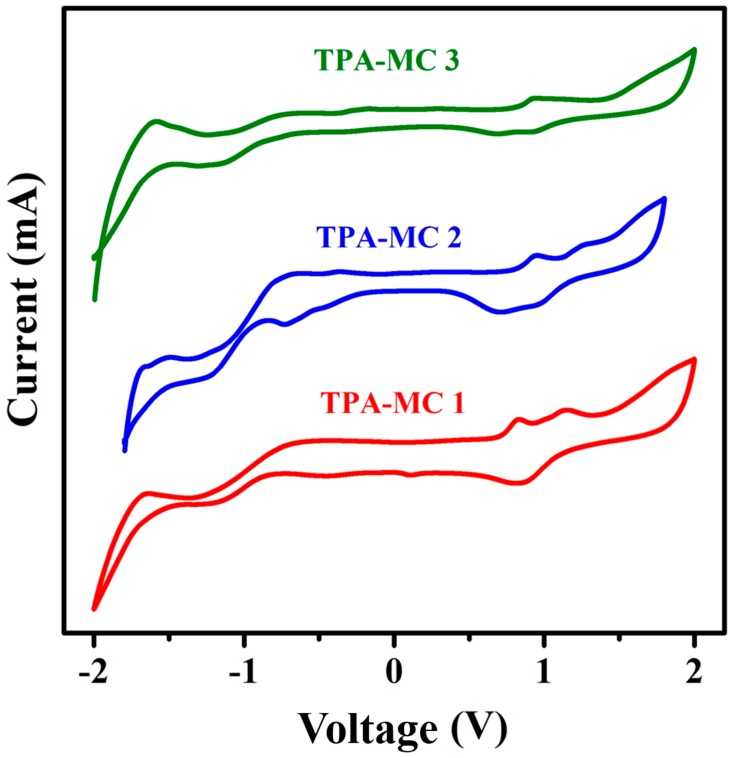
The respective cyclic voltammograms (CV’s) of TPA-MC-1, TPA-MC-2, and TPA-MC-3 recorded from dichlorobenzene solution at a scan rate of 50 µV/s.

**Figure 5:**
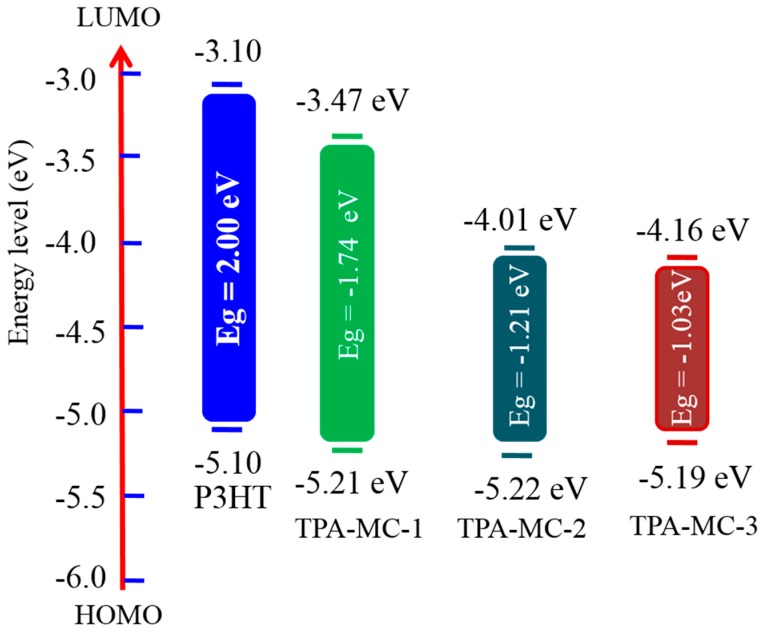
Energy band diagram of P3HT, TPA-MC-1, TPA-MC-2, and TPA-MC-3.

To investigate the electron affinity, semiconductor properties and energy levels of TPA-MC-1, TPA-MC-2, and TPA-MC-3, cyclic voltammetry (CV) measurements [68] in dichlorobenzene were carried out and are depicted in Figure 4; the electrochemical data are summarized in Table 2. The TPA-MC-1 showed the onset oxidation potential at 0.81 V and the onset reduction potential at −0.89 V, which corresponds to the subunits present in the molecular backbone. The first onset oxidation and reduction potentials at 0.82 V and −0.39 V are observed for TPA-MC-2-bearing TCBD, and can be ascribed to donor and acceptor subunits, respectively. For TPA-MC-3, the onset oxidation potential and reduction potential are observed at 0.79 V and −0.24 V, respectively. Relative to TPA-MC-1, the less-negative onset reduction potentials of TPA-MC-2 and TPA-MC-3 are due to the presence of the electron withdrawing TCBD and DCQDCM subunits in the molecular backbone, respectively. The HOMO and LUMO energy levels of TPA-MC-1, TPA-MC-2, and TPA-MC-3 are calculated from the corresponding onset oxidation and reduction potentials. The estimated HOMO/LUMO energy levels of TPA-MC-1, TPA-MC-2, and TPA-MC-3 are −5.21/−3.47 eV, −5.22/−4.01 eV, and −5.19/−4.16 eV, respectively. As compared to TPA-MC-1 the incorporation of TCBD and DCQDCM subunits in backbone of TPA-MC-1, this resulted in a reduction in energy band gap of TPA-MC-2 and TPA-MC-3. The electrochemical properties of TPA-MC-2 and TPA-MC-3 are matches with energy levels of conventional PC71BM (−4.20 eV) acceptor. Moreover, the energy difference between the conventional donor P3HT and the designed acceptors TPA-MC-1, TPA-MC-2, and TPA-MC-3 (Figure 5) is more than 0.3 eV, suggesting the strong electron-withdrawing ability of these new acceptors.

### 2.6. Discussion

Photophysical properties of TPA-MC-1, TPA-MC-2, and TPA-MC-3 were analyzed using UV–Vis electronic absorption and photoluminescence (PL) spectroscopy. All three compounds displayed strong electronic absorption bands at 300–370 nm, which are attributed to the π–π* transition of donor and acceptor units. TPA-MC-1, TPA-MC-2, and TPA-MC-3 compounds showed structured PL spectra at 400–500 nm, suggesting that their excited states exhibit some charge-transfer characteristics. The band gap energies calculated from the onset absorption spectra of TPA-MC-1, TPA-MC-2, and TPA-MC-3 are 1.59 eV, 1.25 eV, and 1.14 eV, respectively. Whereas the band gap energies calculated from DFT calculations showed the same trend: that the energy gap is higher than that of calculated absorption values. The DFT calculation showed in TPA-MC-3, LUMO is completely localized over acceptor units, indicating separated charge HOMO and LUMO charge distributions. The separated HOMO and LUMO electron distribution resulted from the strong electron-donating ability of triphenylamine and strong electron-withdrawing nature of DCQDCM. Furthermore, the HOMO and LUMO measured by CV are 1.74 eV, 1.21 eV, and 1.03 eV for TPA-MC-1, TPA-MC-2, and TPA-MC-3, respectively. UV–Vis and CV results reveal presence of DCQDCM in backbone of the chromophore is more efficient than TCBD for lowering the LUMO level and energy gap. These measured CV energy gap values are comparable with estimated energy gap values by UV–Vis absorption spectroscopy, which confirm their ability to confine as acceptors.

The UV–Vis, PL, CV, and DFT calculation results suggest that TPA-MC-1, TPA-MC-2, and TPA-MC-3 are potential acceptors in for BHJ applications.

## 3. Conclusions

In summary, we designed and synthesized donor–acceptor chromophores TPA-MC-2 and TPA-MC-3 by [2 + 2] cycloaddition–retroelectrocyclization reaction of TPA-MC-1 and TCBD and a DCQDCM, respectively. The LUMO energy level of the TPA-MC-2 and TPA-MC-3 is lowered by incorporation of TCBD and a DCQDCM as strong electron acceptor subunit. The electrochemical properties of TPA-MC-1, TPA-MC-2, and TPA-MC-3 are matches with energy levels of the conventional PC71BM (−4.20 eV) acceptor. Due to the presence of strong electron acceptors in TPA-MC-2, TPA-MC-3 exhibits strong intramolecular charge-transfer (ICT) interactions, which results in lowering of the LUMO energy level. Thus, we believe these along with similar structures will be good candidates for BHJ devices.

## 4. Experimental Section

### 4.1. Materials and Methods

All the chemicals were purchased from Sigma Aldrich, Bengaluru, Karnataka, India. Triphenylamine, 2-(1,3,3-(Trimethylindoline-2-ylidene)acetaldehyde, 4-iodophenyl acetonitrile, triethylamine, copper iodide, (trimethylsilyl)acetylene, bis(triphenylphosphine) palladium (II) chloride, K_2_CO_3_, MgSO_4_, DIPEA, tetrakis (triphenylphosphine) palladium (0), tetracyanoethylene (TCNE), and 7,7,8,8-tetracyanoquinodimethane (TCNQ) were used as-received without further purification unless otherwise mentioned. The moisture sensitized reactions were carried out under nitrogen atmosphere using freeze-thaw-pump cycle method. Solvents were purchased from SD Fine, India and are AR grade. The solvents were distilled before use. The structure of prepared compounds was confirmed by using modern spectroscopic techniques. The progress of reactions was monitored by TLC. The TLC results were visualized by a UV light (254 or 356 nm). IR spectra were recorded on Thermo Nicolet Nexus 670. ^1^H NMR (300 MHz and 400 MHz) spectra and ^13^C NMR (75 MHz and 100 MHz) were measured at 298 K using CDCl_3_ as a solvent. Tetramethylsilane (δ = 0 ppm) was used as an internal standard. ESI-MS data were taken on Shimadzu lab solution mass spectrometer. High-resolution mass spectra (HRMS) spectrometry (Thermofisher Exactive Orbitrap) and atmospheric-pressure chemical ionization (APCI) experiments were carried out on Fourier-transform mass spectroscopy (FTMS). UV–Vis absorption was recorded on a UV–Vis-1800 spectrometer (Shimadzu, Japan) in CHCl_3_ solvent and thin film on quartz surface at room temperature. Florescence spectra were recorded on R-6000 spectrofluorophotometer (Shimadzu, Japan) in CHCl_3_ solvent. Thermal stability was analyzed by thermogravimetric analysis (TGA) at the heating rate 10 °C per min under nitrogen atmosphere. The cyclic voltammograms were measured on Power Lab ML160 potentiostat interfaced via a Power Lab 4/20 controller to a PC running E-Chem for Windows version 1.5.2 electrochemical analyzer. The cyclic voltammetry experiments were performed using tetra butyl ammonium hexaflurophospate (TBAPF_6_, 1.0 M) as supporting electrolyte, Pt as a working electrode, Pt wire used as a counter electrode and saturated calomel electrode (SCE) (Ag/AgCl in saturated KCl) used as a reference electrode.

### 4.2. Synthetic Procedure of Compound ***1***

In a 250-mL round bottom flask, triphenylamine (3.00 g, 12.2 mmoL) was dissolved in the mixture of chloroform (60 mL) and acetic acid (60 mL) in the same equimolar ratio (1:1, *v/v*). The N-iodosuccinamide (2.75 g, 12.2 mmoL) was added and then reaction mixture was stirred at room temperature about 12 h under dark condition. The completion of reaction was confirmed by TLC. The reaction mixture was poured into water and extracted with CHCl_3_. The organic layer was washed with water. The excess iodine was quenched with saturated Na_2_S_2_O_3_. The obtained colorless organic layer was dried over sodium sulfate. The combined organic layer was concentrated under reduced pressure. The residue, colorless oily liquid, was dried under vacuum. The crude product was washed with hexane (2–3 times) to yield white powder as **1** (4.17 g, 92%). ^1^H NMR (400 MHz, CDCl_3_) δ 7.51 (d, *J* = 8.8 Hz, 2H), 7.24 (t, *J* = 8.4 Hz, 4H), 7.106.99 (m, 4H), 6.82 (d, *J* = 8.9 Hz, 2H).

### 4.3. Synthetic Procedure of Compound ***2***

(**a**) A mixture of compound **1** (3 g, 8.08 mmoL), ethynyltrimethylsilane (3.36 mL, 24.2 mmoL), and CuI (76 mg, 0.40 mmol) were dissolved in mixture of triethylamine (30 mL) and dry THF (30 mL). The Pd(PPh_3_)_2_Cl_2_ (ca. 5mol%) was added to the reaction mixture under nitrogen atmosphere. The resulting mixture was refluxed for 12 h under nitrogen atmosphere. The progress of reaction was monitored by TLC. After completion of reaction, the reaction mixture was poured into 200 mL of water and extracted with DCM (2–3 times). The combined organic layer was dried over anhydrous Na_2_SO_4_. The solvent was concentrated under reduced pressure to yield **2** as yellow oil. The obtained crude product was used in next step without purification.

(**b**) Compound **2** was dissolved in the mixture of 30 mL of THF and 30 mL of methanol. The dry fine powder of K_2_CO_3_ (2.6 g) was added to the reaction mixture. The resulting mixture was stirred for 4 h at room temperature. The completion of reaction was monitored by TLC. The solvent was evaporated under reduced pressure. The obtained crude product was diluted with DCM and filtered off. The organic solvent was concentrated under reduced pressure. The obtained crude product was purified by column chromatography on silica gel to afford light yellow solid **3** (yield, 70%). ^1^H NMR (400 MHz, CDCl_3_) δ 7.34–7.30 (m, 2H), 7.297.24 (m, 4H), 7.107.03 (m, 6H), 7.986.94 (m, 2H), 3.01 (s, 1H); ^13^C NMR (125 MHz, CDCl_3_) δ 148.33, 147.09, 133.03, 129.38, 125.02, 123.60, 122.02, 114.72, 76.14.

### 4.4. Synthetic Procedure of Compound ***6***

4-Iodo phenyl acetonitrile **4** (1 g, 4.1 mmoL) was dissolved in freshly prepared dry THF (20 mL). Piperidine (1 mL) was added to this reaction mixture under nitrogen atmosphere. The resulting reaction mixture was refluxed for 1 h. The 2-(1, 3, 3-trimethylindolin-2-ylidene) acetaldehyde **5** (1 g, 4.9 mmoL) was added to the reaction mixture at refluxed temperature and continued the reaction for 12 h. The progress of reaction was monitored with TLC. After completion of reaction, the excess aldehyde was washed with chilled methanol. This process was performed till the complete removal of aldehyde. The obtained product was dried over vacuum to yield shiny dark yellow compound **6** (1.50 g, yield: 86%). FT-IR (in KBr, cm^−1^): 3050.13, 2966.11, 2192.35, 1589.82, 1564.09, 14484.37, 1334.73, 1203.73, 1120.90, 981.30, 746.59, 706.20 cm^−1^; ^1^H NMR (400 MHz, CDCl_3_) δ 7.82 (d, *J* = 12.7 Hz, 1H), 7.68 (d, *J* = 8.6 Hz, 2H), 7.27–7.20 (m, 4H), 6.98 (t, *J* = 7.4, 6.7 Hz, 1H), 6.79 (d, *J* = 7.9 Hz, 1H), 5.87 (d, *J* = 12.7 Hz, 1H), 3.29 (s, 3H), 1.63 (s, 6H); ^13^C NMR (100 MHz, CDCl_3_) δ 164.86, 144.05, 138.90, 137.89, 135.06, 128.15, 125.98, 121.62, 121.40, 118.49, 107.32, 99.40, 94.40, 91.25, 46.65, 29.57, 28.83. ESI–Mass m/z 427 [M+H]^+^; HRMS: Chemical Formula: C_21_H_19_IN_2_ m/z calculated 427.0646 [M+H]^+^, found: 427.0665 [M+H]^+^.

### 4.5. Synthetic Procedure of TPA-MC-1

The mixture of dry THF (10 mL) and dry DIPEA (10 mL) was deoxygenated under nitrogen atmosphere for 30 min. Compound **6** (500 mg, 1.17 mmoL) and compound **3** (347 mg, 1.29 mmoL) were added to the reaction mixture. The resulting reaction mixture was carefully degassed and recharged with N_2_ gas. A catalytic amount of tetrakis (triphenylphosphine)palladium (0) (ca. 5 moL %) and copper iodide (12 mg, 0.05 mmoL) were added simultaneously. The resulting reaction mixture was stirred under reflux for 12 h. The completion of reaction was monitored with TLC. The reaction mixture was cooled to room temperature. The solvent was removed under reduced pressure. The obtained residue was dissolved in excess DCM and passed through celite. The obtained filtrate was concentrated under reduced pressure. The crude product was purified by column chromatography on silica gel (60/120 mesh) with dichloromethane/hexane (3: 6) as an eluent to afford TPA-MC-1 as a dark orange solid (546 mg, yield: 82%). FT-IR (in KBr, cm^−1^): 3447.42, 3031.13, 2923.17, 2195.35, 1572.15, 1510.82, 1487.41, 1386.64, 1332.91, 1273.40, 1199.75, 1120.04, 1075.10, 924.71, 814.64, 746.63, 694.14, 514.69 cm^−1^; ^1^H NMR (400 MHz, CDCl_3_) δ 7.86 (d, *J* = 12.7 Hz, 1H), 7.507.45 (m, 4H), 7.397.36 (m, 2H), 7.297.20 (m, 7H), 7.136.96 (m, 9H), 6.79 (d, *J* = 7.9 Hz, 1H), 5.91 (d, *J* = 12.7 Hz, 1H), 3.30 (s, 3H), 1.65(s, 6H); ^13^C NMR (100 MHz, CDCl_3_) δ 164.64, 147.88, 147.15, 144.13, 139.02, 138.64, 134.57, 132.47, 131.91, 131.63, 129.36, 128.54, 128.08, 124.95, 124.00, 123.52, 122.28, 121.67, 121.37, 118.63, 116.04, 107.34, 100.11, 94.61, 90.66, 88.70, 46.65, 29.59, 28.87, 28.36; ESI–Mass m/z 568 [M+H]^+^; HRMS: Chemical Formula: C_41_H_33_N_3_ calculated m/z 567.2666 [M]^+^, found: 567.2669 [M]^+^.

### 4.6. Synthetic Procedure of TPA-MC-2

The compound TPA-MC-1 (100 mg, 0.17 mmoL) was dissolved in dry dichloromethane (5 mL). To this reaction mixture, tetracyanoethylene (33 mg, 0.26 mmoL) was added. After addition of TCNE, immediate color change of reaction mixture was observed. The progress of reaction was monitored by TLC. After 2 h, the starting material was completely consumed. The solvent was concentrated under reduced pressure and the residue was purified by column chromatography on silica gel with DCM/hexane (8:2) as eluent to get dark purple color solid TPA-MC-2 (116 mg, 95%). FT-IR (in KBr, cm^−1^): 2923.82, 22, 2218.20, 2197.17, 1587.61, 1562.93, 1485.71, 1426.26, 1338.47, 1288.33, 1256.41, 1203.32, 1175.43, 1118.93, 1049.24, 930.55, 816.00, 749.34, 698.03, 522.2 cm^−1^; ^1^H NMR (400 MHz, CDCl_3_) δ 8.05 (d, *J* = 12.9 Hz, 1H), 7.79 (d, *J* = 8.9 Hz, 2H), 7.68 (d, *J* = 9.2 Hz, 2H), 7.62 (d, 8.9 Hz, 2H), 7.427.38 (m, 4H), 7.327.27 (m, 4H), 7.247.21 (m, 5H), 7.097.05 (m, 1H), 6.94 (d, *J* = 9.2 Hz, 2H), 6.89 (d, *J* = 7.9 Hz, 1H), 3.38 (s, 3H), 1.66 (s, 6H).^13^C NMR (100 MHz, CDCl_3_) δ 167.89, 166.44, 164.20, 153.73, 144.48, 143.50, 141.64, 139.36, 131.85, 130.46, 130.06, 128.33, 126.92, 126.68, 124.51, 122.68, 121.86, 121.54, 118.04, 117.81, 113.58, 112.76, 112.95, 108.26, 97.40, 95.49, 83.11, 47.43, 29.95, 29.67, 28.88; ESI–Mass m/z 696 [M]^+^; HRMS: Chemical Formula: C_47_H_33_N_7_ calculated m/z 696.2906 [M+H]^+^, found: 696.2870 [M+H]^+^.

### 4.7. Synthetic Procedure of TPA-MC-3

To a solution of TPA-MC-1 (100 mg, 0.17 mmoL) in dry THF (5 mL), TCNQ (53 mg, 0.26 mmol) was added. The resulting reaction mixture was refluxed for 24 h. The progress of reaction was monitored by TLC. After completion of reaction, the solvent was evaporated under reduced pressure. The obtained crude product was purified by column chromatography on silica to yield black solid TPA-MC-3 (121 mg, 89%). FT-IR (in KBr, cm^−1^): 2923.08, 2205.44, 1556.92, 1488.49, 1413.25, 1386.02, 1317.82, 1178.999, 1119.71, 1019.22, 923.54, 835.07, 809.83, 748.78, 697.30, 522.86 cm^−1^; ^1^H NMR (400 MHz, CDCl_3_) δ 8.02–7.96 (m, 1 H), 7.71–7.70 (m, 2 H), 7.62–7.53 (m, 4 H), 7.39–7.35 (m, 5 H), 7.31–7.17 (m, 11 H), 7.08–7.03(m, 1H), 6.89–6.84 (m, 2 H), 5.98 (d, *J* = 12.9 Hz, 1 H), 3.37–3.36 (m, 3 H), 1.66–1.65 (m, 6 H); ^13^C NMR (100 MHz, CDCl_3_) δ 170.08, 167.56, 166.71, 154.02, 151.54, 151.01, 145.32, 144.69, 143.54, 141.72, 141.27, 140.43, 139.26, 135.23, 134.24, 133.33, 132.69, 132.12, 131.08, 130.67, 129.87, 128.33, 126.86, 126.55, 125.83, 125.53, 124.43, 122.58, 121.82, 119.28, 117.91, 114.08, 113.52, 112.78, 108.19, 97.79, 95.33, 84.00, 74.21, 47.34, 29.92, 28.89, 14.10; ESI–Mass m/z 772 [M+H]^+^; HRMS: Chemical Formula: C_47_H_33_N_7_ calculated m/z 772.3212 [M+H]^+^, found: 772.3183 [M+H]^+^.

## Figures and Tables

**Table 1:** Optical properties of TPA-MC-1, TPA-MC-2 and TPA-MC-3.

Acceptor	λ_max_ Solution (nm)	λ_max_ Film (nm)	λ^onset^ Value Film (nm)	Optical Bandgap *E_g_* (eV) ^a^
TPA-MC-1	442, 364, 313	445, 315	776	1.59
TPA-MC-2	595, 481, 282 (Shoulder)	631, 498, 300	986	1.25
TPA-MC-3	663, 483, 291	670, 485, 300	1083	1.14

^a^ Optical band gap estimated from onset wavelength of film (λ_edge_) of the absorption spectra of thin film: *E*_g_^opt^ = 1240/λ_edge_.

**Table 2:** Electrochemical properties of TPA-MC-1, TPA-MC-2 and TPA-MC-3.

Acceptor	E_ox_^onset^ (V)	E_red_^onset^ (V)	HOMO = [E_red_^onset^ + 4.8] (eV)	LUMO = [E_red_^onset^ + 4.8] (eV)	E_g_ = (HOMO-LUMO) eV
TPA-MC-1	0.81	−0.89	−5.21	−3.47	1.74
TPA-MC-2	0.82	−0.39	−5.22	−4.01	1.21
TPA-MC-3	0.79	−0.24	−5.19	−4.16	1.03

## Supplementary Materials

Supplementary materials can be found at https://www.mdpi.com/1422-0067/20/7/1621/s1.

## Supporting Information

TGA, Molecular orbital diagrams, calculated TA-DFT, computed absorption spectras, ^1^H NMR, ^13^C NMR, FT-IR, mass and HRMS are given in SI.

## Abbreviations

CV	Cyclic Voltammetry
DCQDCM	Dicyanoquinodicyanomethane
HOMO	Highest occupied molecular orbital
ICT	Intramolecular charge-transfer
LUMO	Lowest unoccupied molecular orbital
MC	Merocyanine
TCBD	1,1,4,4-tetracyanobutadiene
TCNE	Tetracyanoethene
TCNQ	7.7.8,8-tetracyanoquinodimethane
TPA	Triphenylamine
UV–Vis	Ultraviolet Visible
