# Supplementary material for: Triphenylamine-Merocyanine-Based D1-A1-π-A2/A3-D2 Chromophore System: Synthesis, Optoelectronic, and Theoretical Studies
Source: Int J Mol Sci. 2019 Apr 1;20(7):1621. doi: 10.3390/ijms20071621 (PMC6479914; doi:10.3390/ijms20071621)
Supplement: Supplementary file 1 [file ijms-20-01621-s001.pdf]

# Supplementary Information

## Triphenylamine-merocyanine based D1-A1-π-A2/A3-D2 chromophore system: Synthesis, optoelectronic and theoretical studies

**Pedada Srinivasa Rao[1,2], Avinash L. Puyad[3], Sidhanath V. Bhosale[1,2]* and Sheshanath V. Bhosale[4]***

[1]Polymers and Functional Materials Division, CSIR-Indian Institute of Chemical Technology, Hyderabad 500007, Telangana, India.
[2]Academy of Scientific and Innovative Research (AcSIR), Ghaziabad-201002, India
[3]School of Chemical Sciences, Swami Ramanand Teerth Marathwada University, Nanded-431606, Maharashtra, India
[4]Department of Chemistry, Goa University, Taleigao Plateau, Goa-403206, India
* Correspondence: bhosale@iict.res.in; svbhosale@unigoa.ac.in

**Keywords:** donor-acceptor; cyclic voltammograms; triphenylamine; tetracyanoethylene; 7,7,8,8-tetracyanoquinodimethane

---

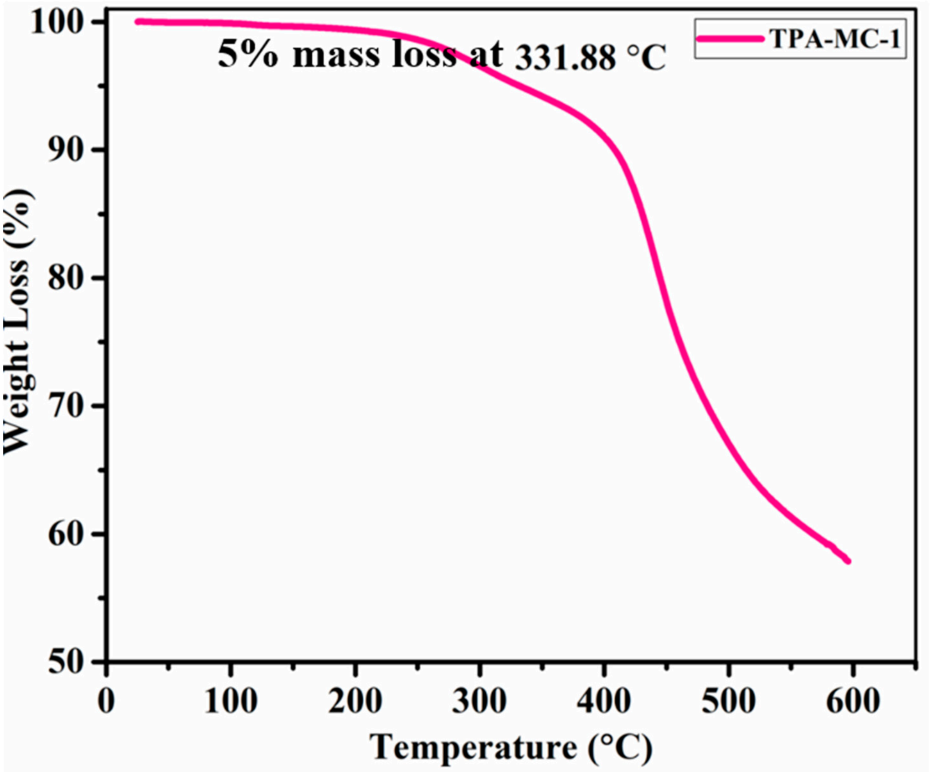


**Figure S1.** Thermogravimetric analysis (TGA) graph of TPA-MC-1.

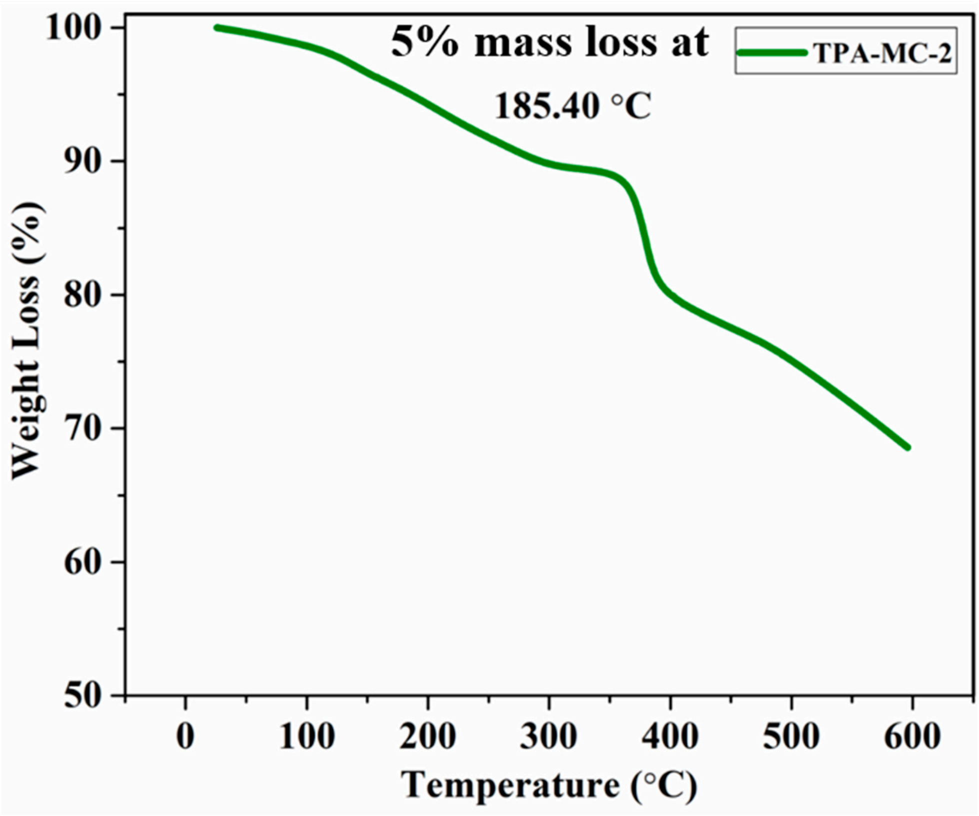


**Figure S2.** Thermogravimetric analysis (TGA) graph of TPA-MC-2.

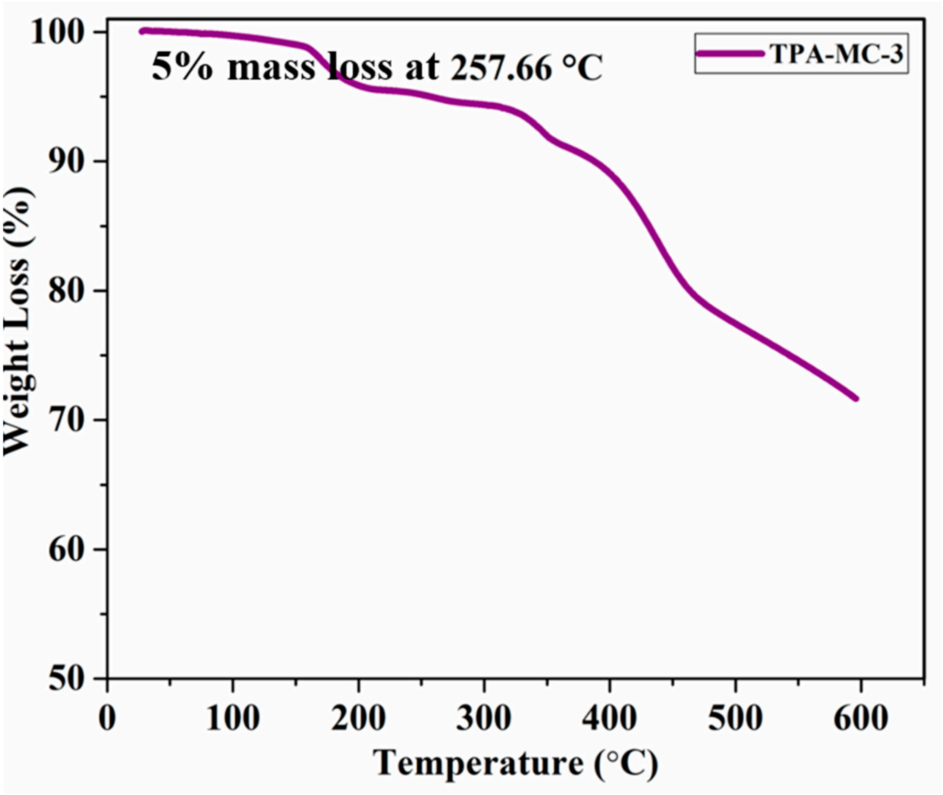


**Figure S3.** Thermogravimetric analysis (TGA) graph of TPA-MC-3.

**Table S1**. Calculated TD-DFT excitation properties of TPA-MC-1, TPA-MC-2 and TPA-MC-3

| Molecules | Excitation Energy (eV) | Excitation Wavelength (nm) | Oscillator Strength (f) | Excitations | Percentage contribution for transition |
|---|---|---|---|---|---|
| **TPA-MC-1** | 2.5831 | 479.98 | 2.1566 | 150 ->151 | HOMO->LUMO (98%) |
| **TPA-MC-2** | 1.8305 | 677.34 | 0.5899 | 182 ->183 | HOMO->LUMO (96%) |
| | | | | 182 ->184 | HOMO->L+1 (3%) |
| | 2.2915 | 541.06 | 0.7965 | 182 ->183 | HOMO->LUMO (3%) |
| | | | | 182 ->184 | HOMO->L+1 (93%) |
| | 2.6517 | 467.57 | 0.6391 | 181 ->183 | H-1->LUMO (4%) |
| | | | | 181 ->184 | H-1->L+1 (93%) |
| **TPA-MC-3** | 1.4538 | 852.85 | 1.0404 | 202 ->203 | HOMO->LUMO (98%) |
| | 2.2895 | 541.52 | 0.9685 | 200 ->203 | H-2->LUMO (50%) |
| | | | | 202 ->204 | HOMO->L+1 (44%) |
| | 2.6822 | 462.25 | 0.6986 | 199 ->203 | H-3->LUMO (5%) |
| | | | | 200 ->203 | H-2->LUMO (2%) |
| | | | | 201 ->204 | H-1->L+1 (85%) |
| | | | | 202 ->205 | HOMO->L+2 (5%) |

| **TPA-MC-1** | | **eV** | |
|---|---|---|---|
| L | 151 | -1.90127 | 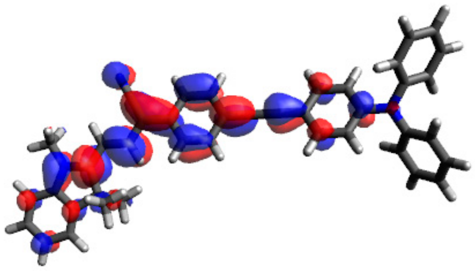 |

| | | | |
|---|---|---|---|
| H | 150 | -4.81428 | 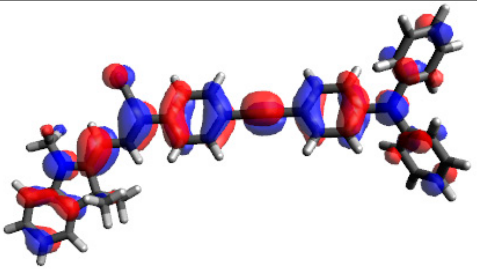 |
| **TPA-MC-2** | | | |
| 184 | L+1 | -2.64034 | 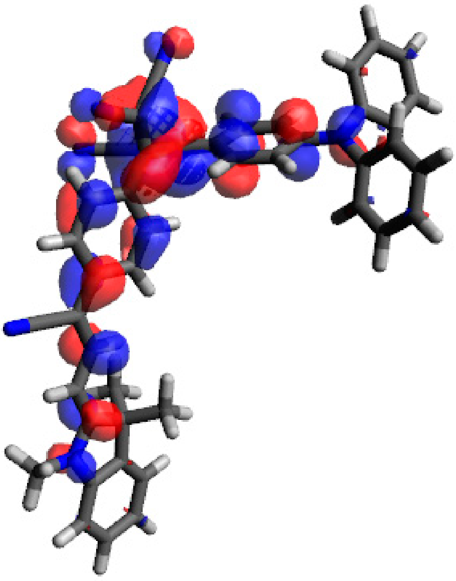 |
| 183 | L | -3.15355 | 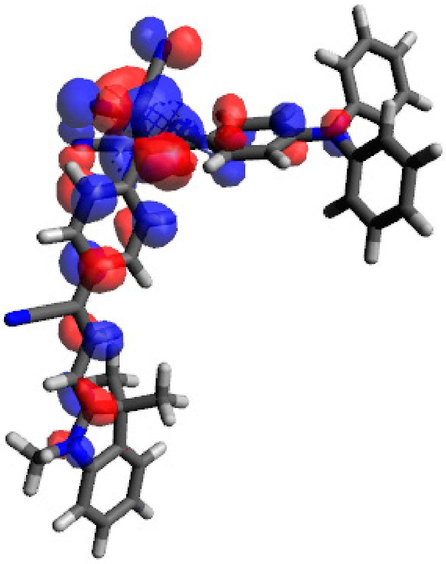 |

| | | | |
|---|---|---|---|
| 182 | H | -5.25919 | 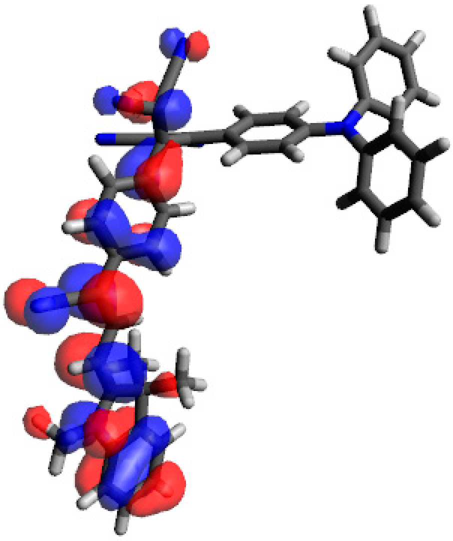 |
| 181 | H-1 | -5.60641 | 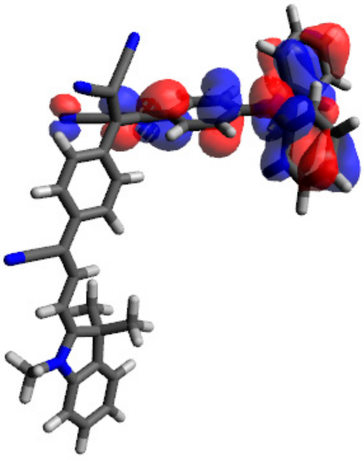 |
| **TPA-MC-3** | | | |
| 205 | L+2 | -1.93175 | 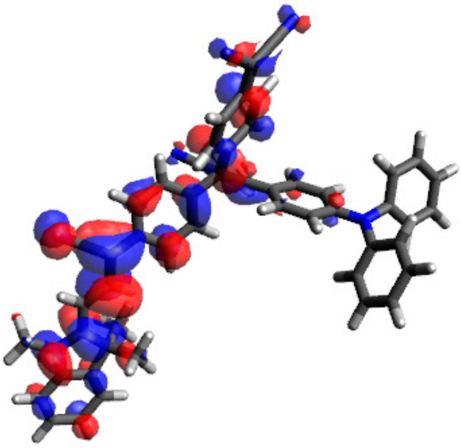 |

| | | | |
|---|---|---|---|
| 204 | L+1 | -2.5519 | 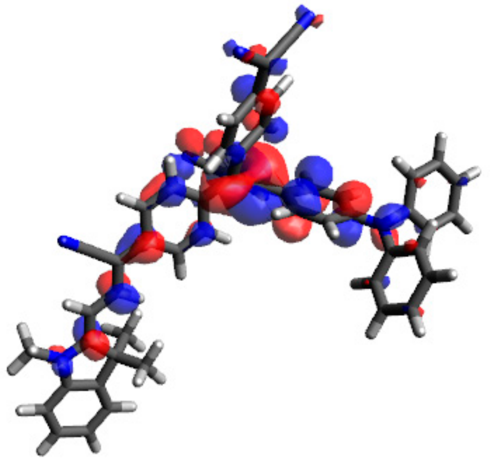 |
| 203 | L | -3.57397 | 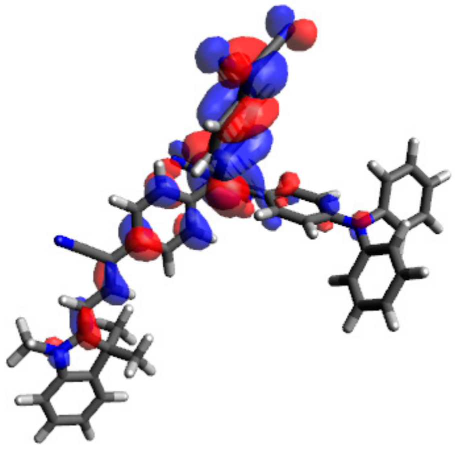 |
| 202 | H | -5.18 | 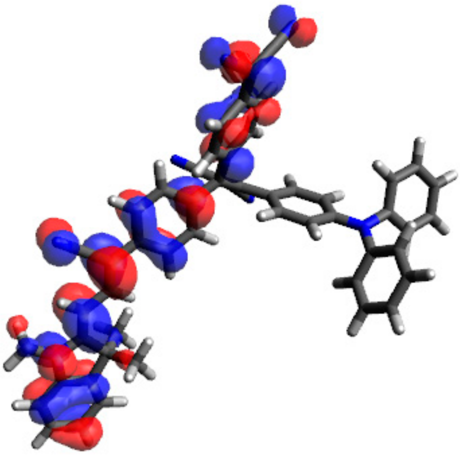 |

| | | | |
|---|---|---|---|
| 201 | H-1 | -5.56804 | 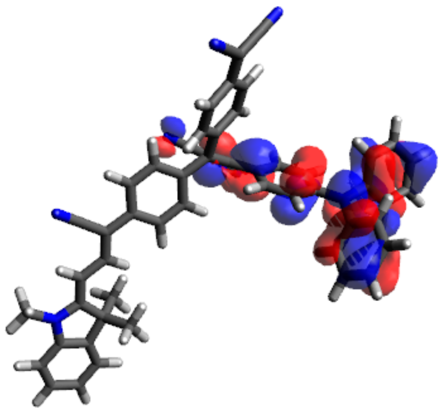 |
| 200 | H-2 | -5.96533 | 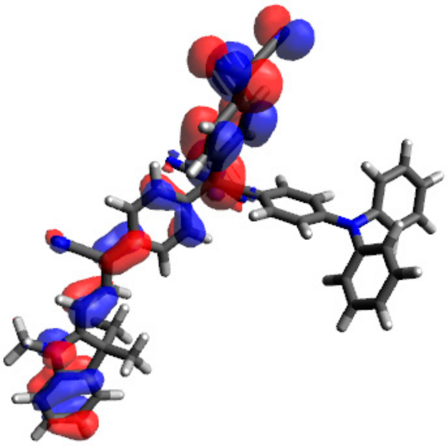 |
| 199 | H-3 | -6.78929 | 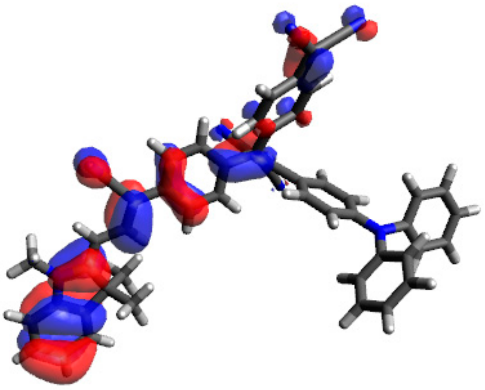 |

**Figure S4.** Frontier molecular orbitals of TPA-MC-1, TPA-MC-2 and TPA-MC-3 with energy in eV**.**

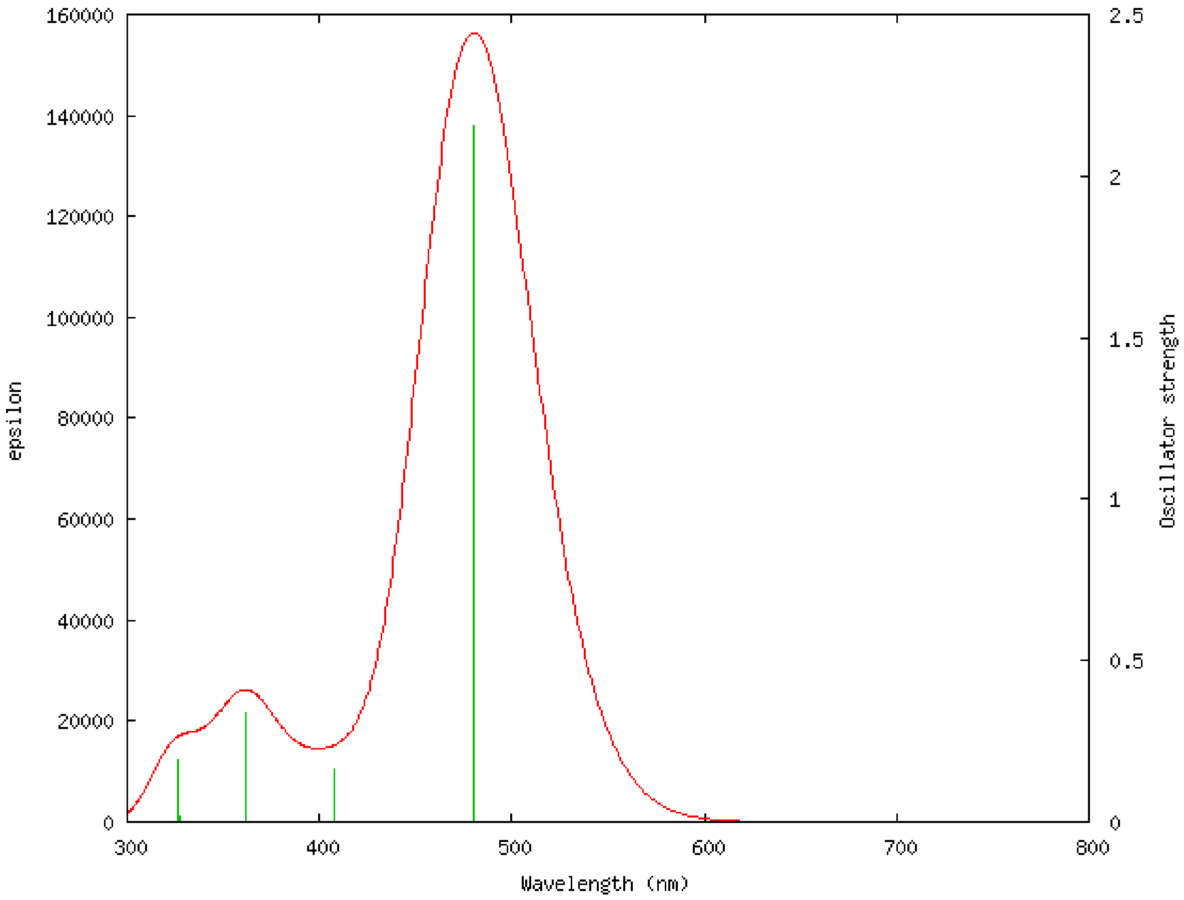


**Figure S5.** The computed absorption spectra of TPA-MC-1.

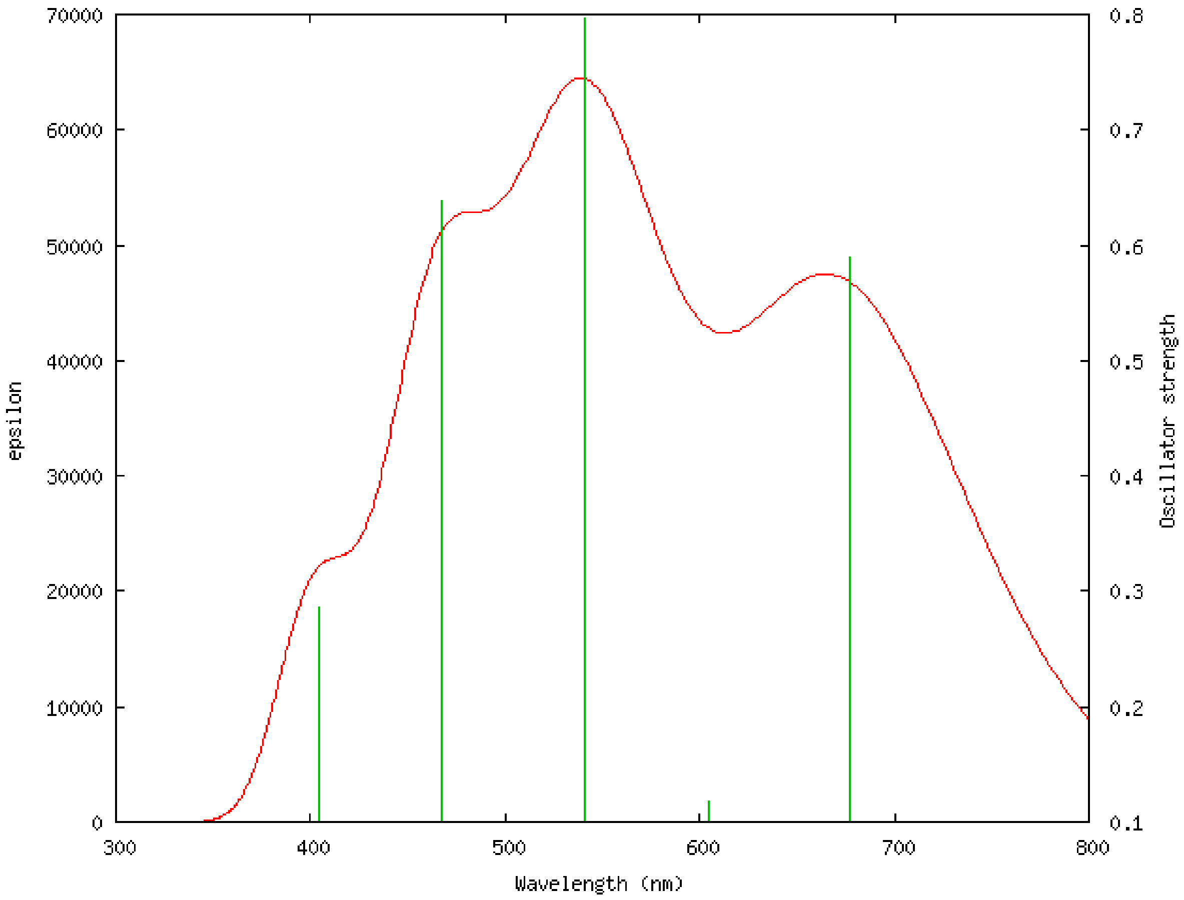


**Figure S6.** The computed absorption spectra of TPA-MC-2.

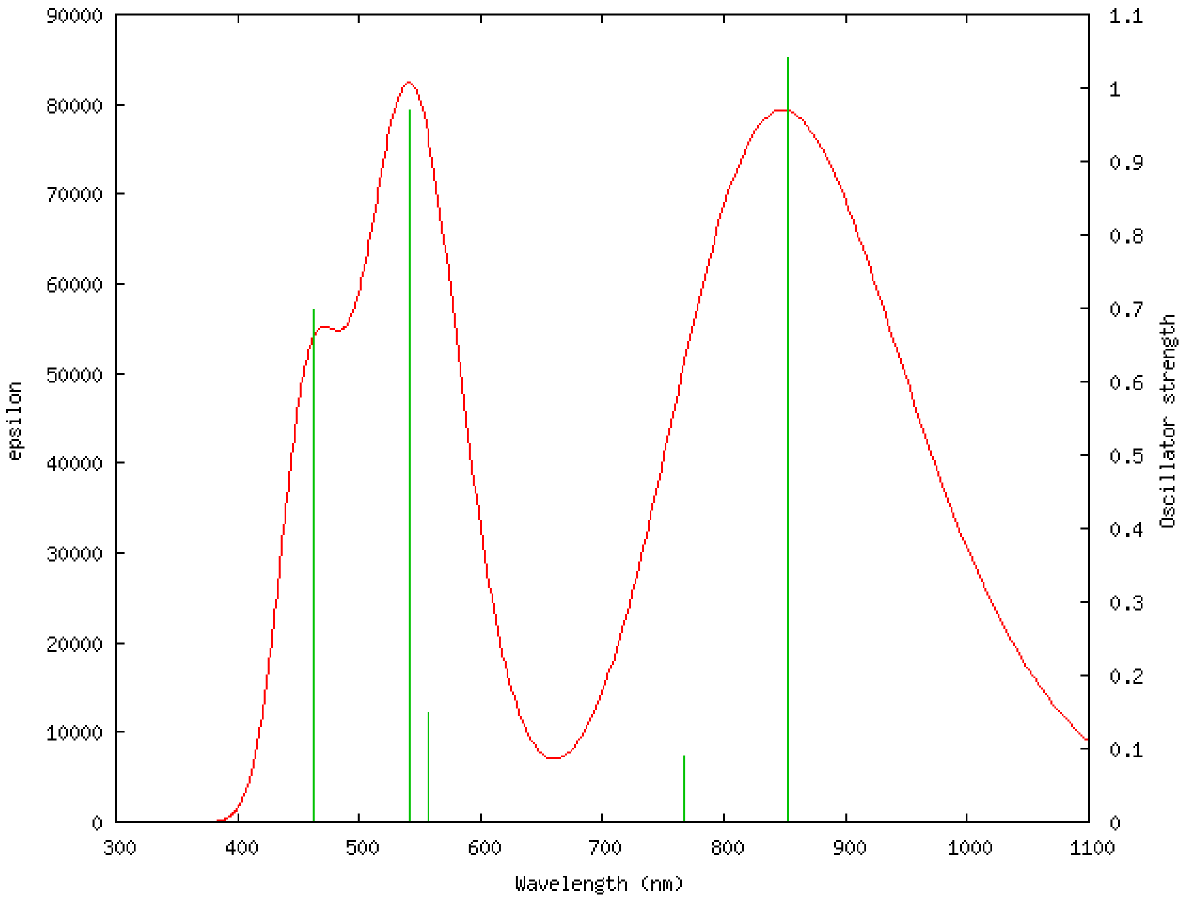


**Figure S7.** The computed absorption spectra of TPA-MC-3.

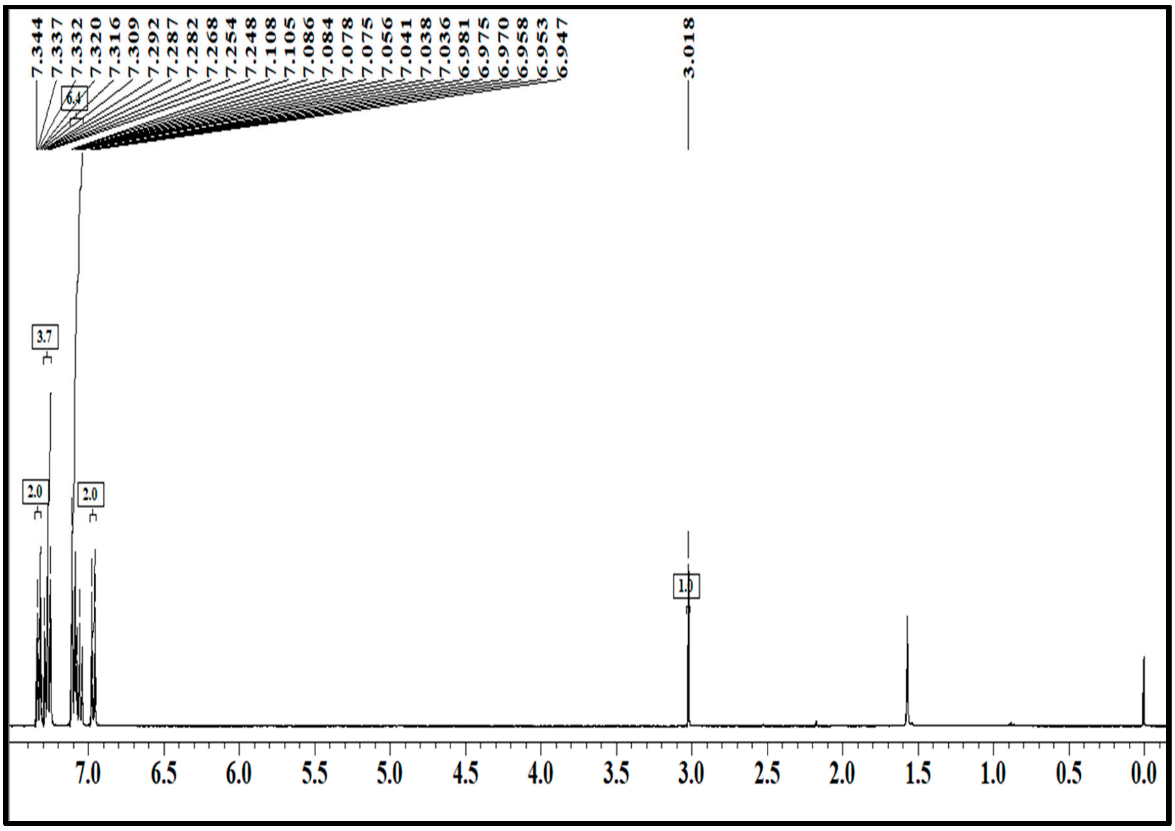

**Figure S8.** $^{1}$H NMR spectra of compound 3.

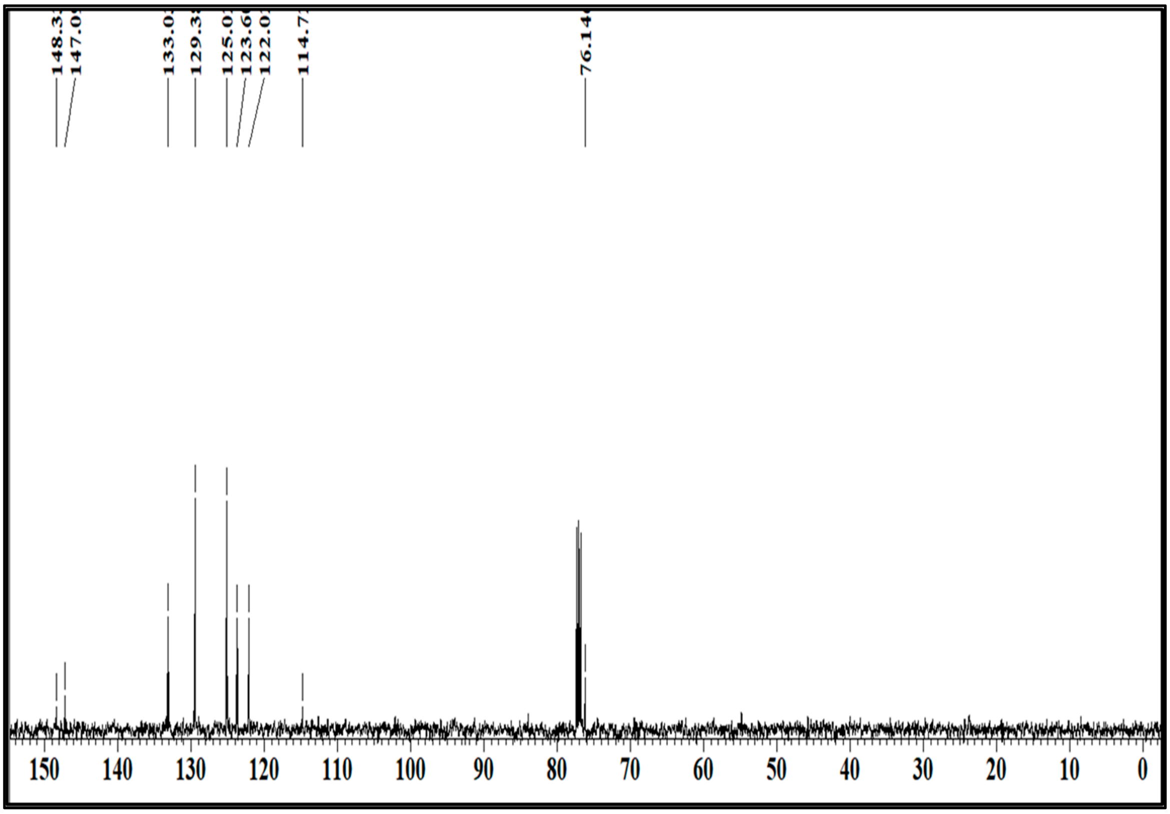


**Figure S9.** $^{13}C$ NMR spectra of compound 3.

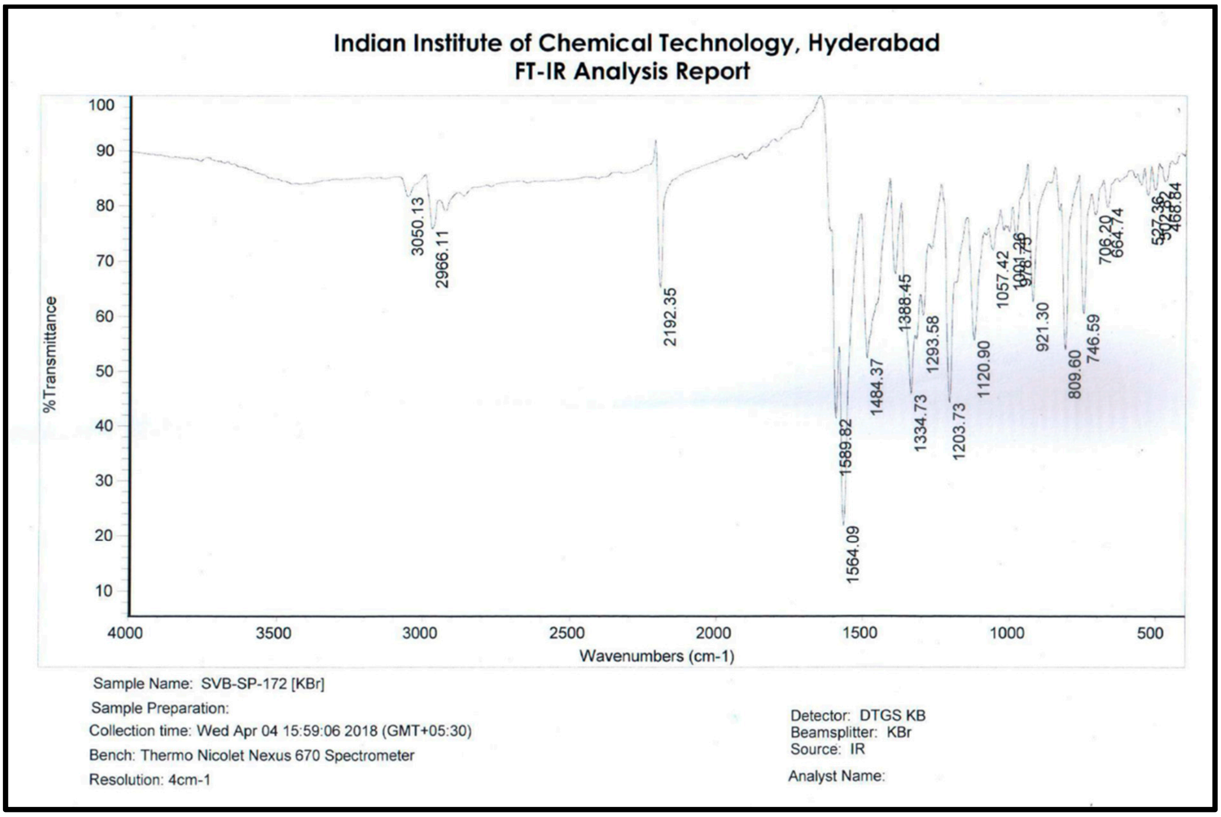


**Figure S10.** FT-IR spectra of compound 4.

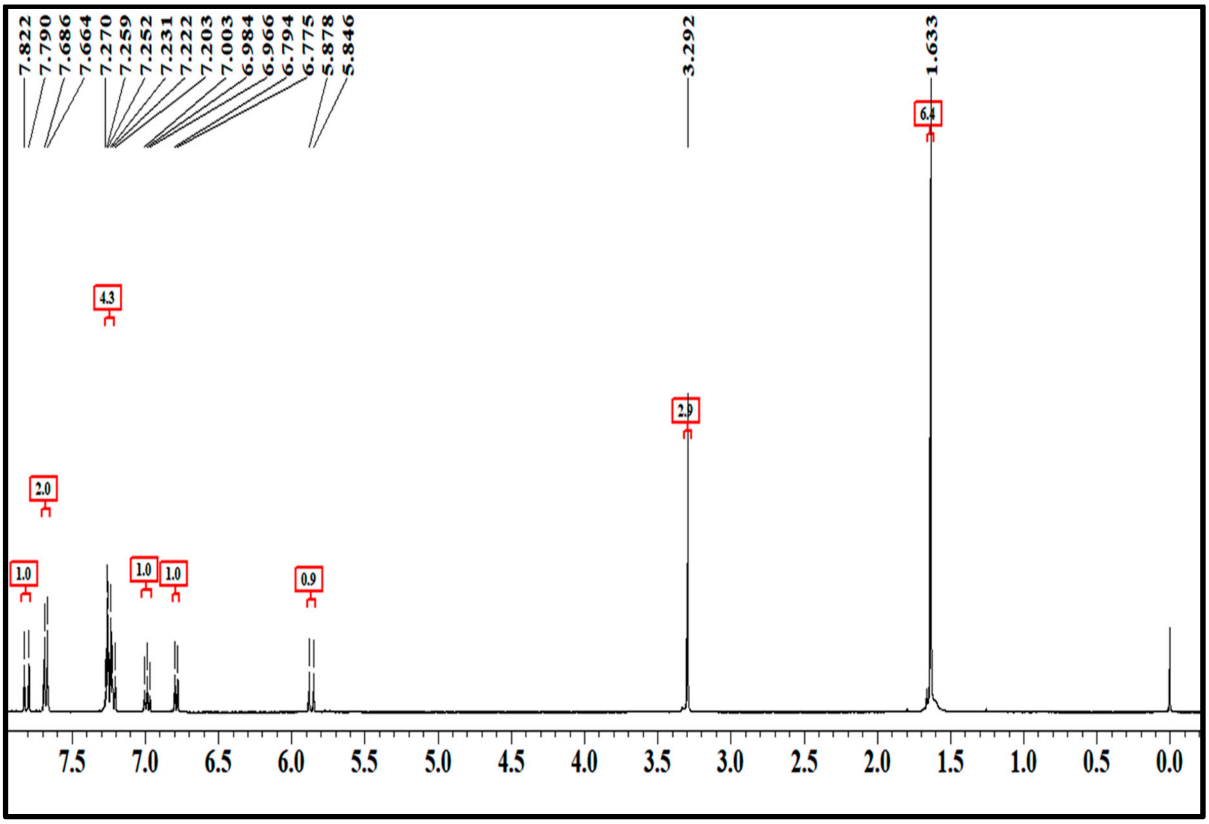


**Figure S11.** $^{1}$H NMR spectra of compound 4.

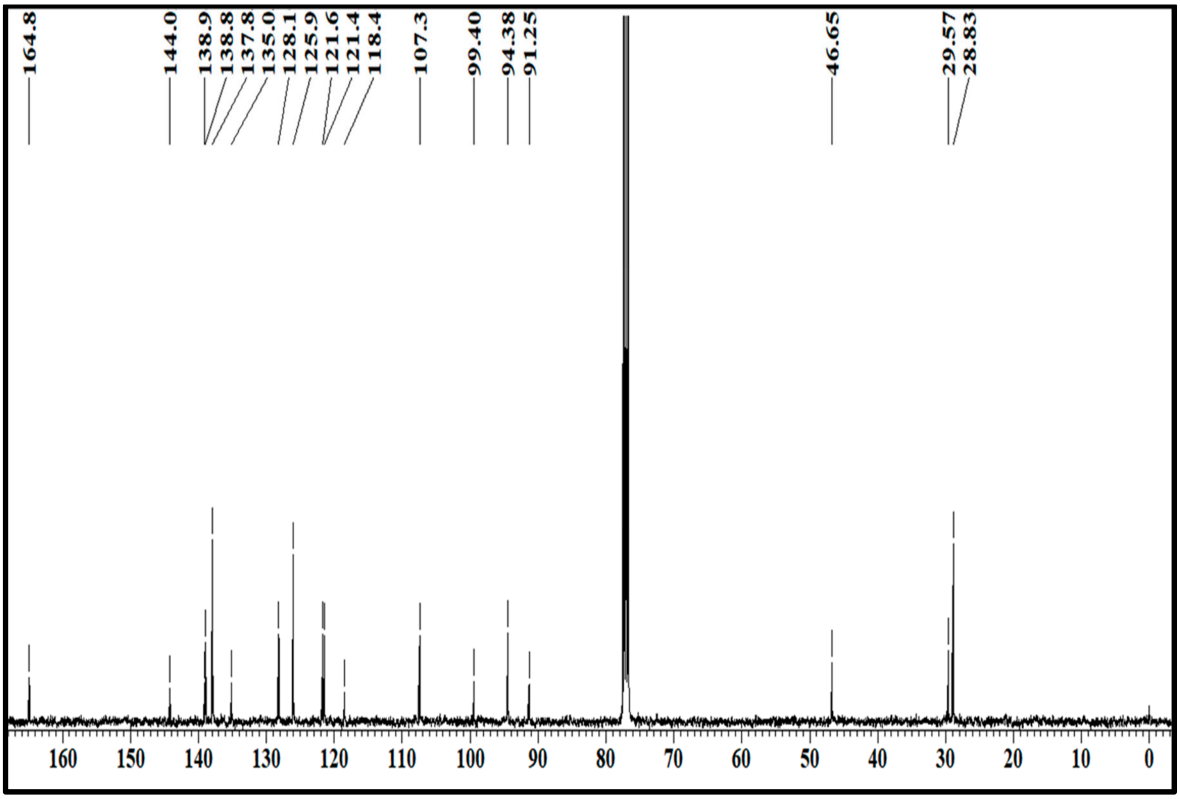


**Figure S12.** $^{13}$C NMR spectra of compound 4.

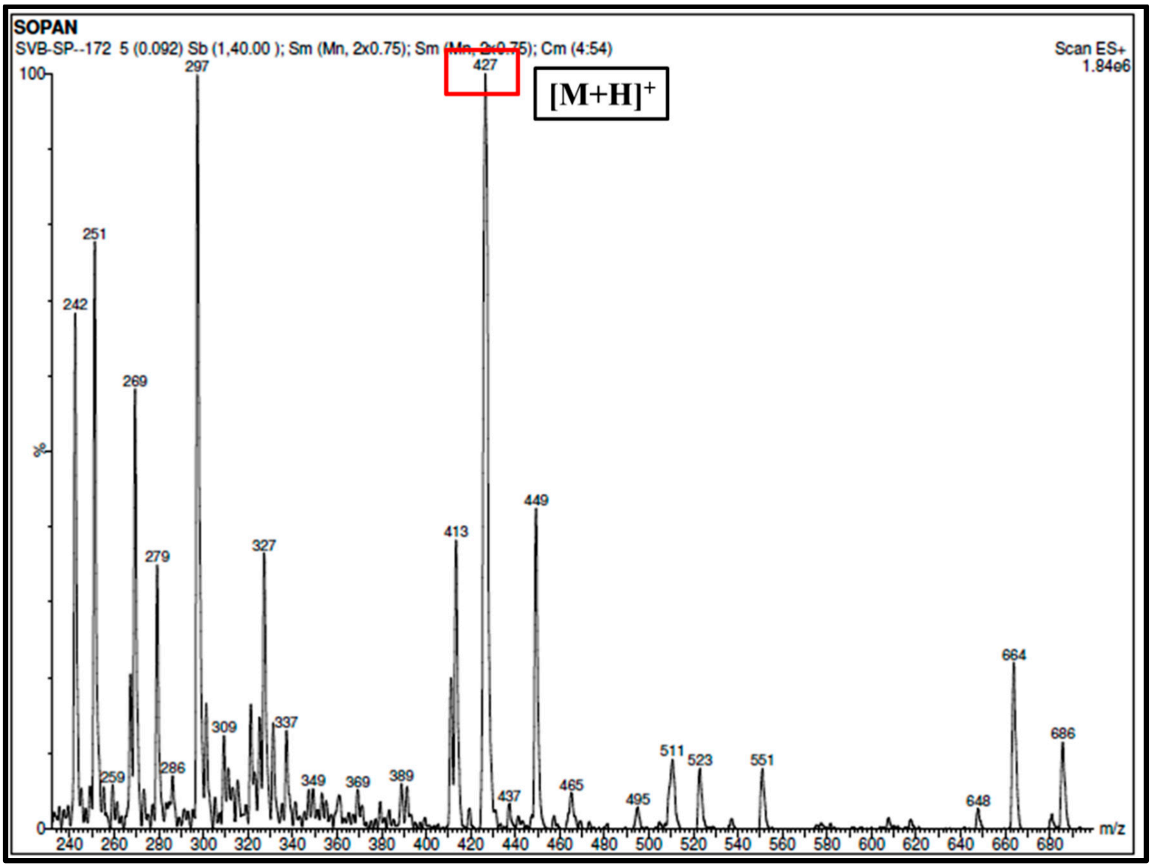


**Figure S13.** ESI-Mass of Compound 4.

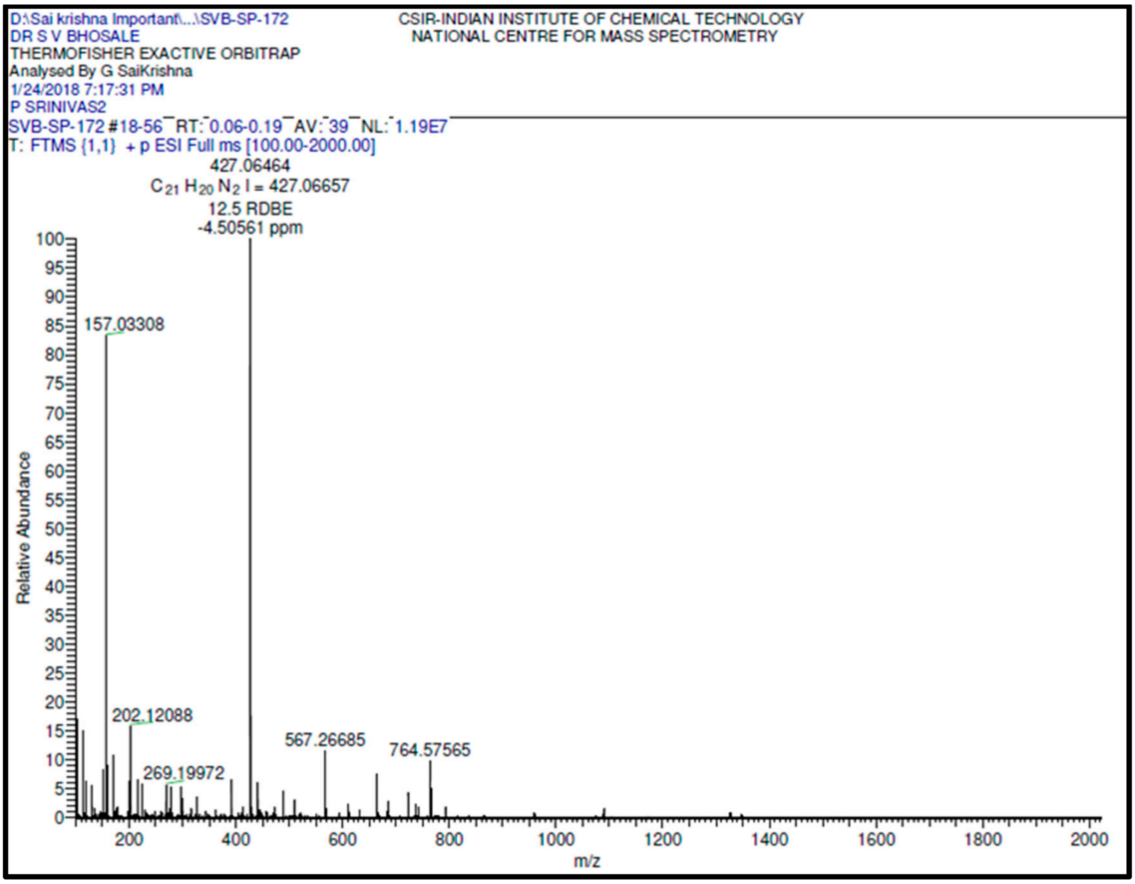

**Figure S14.** HRMS of Compound 4.

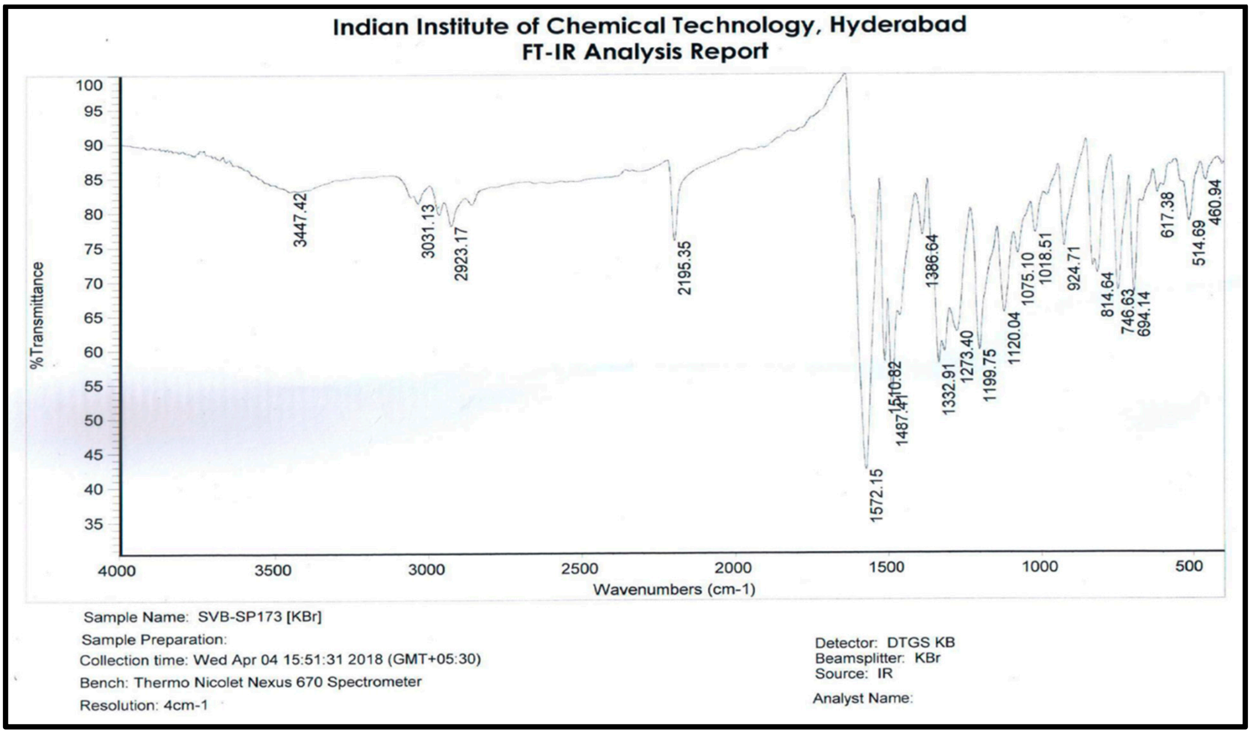


**Figure S15.** FT-IR spectra of compound TPA-MC-1.

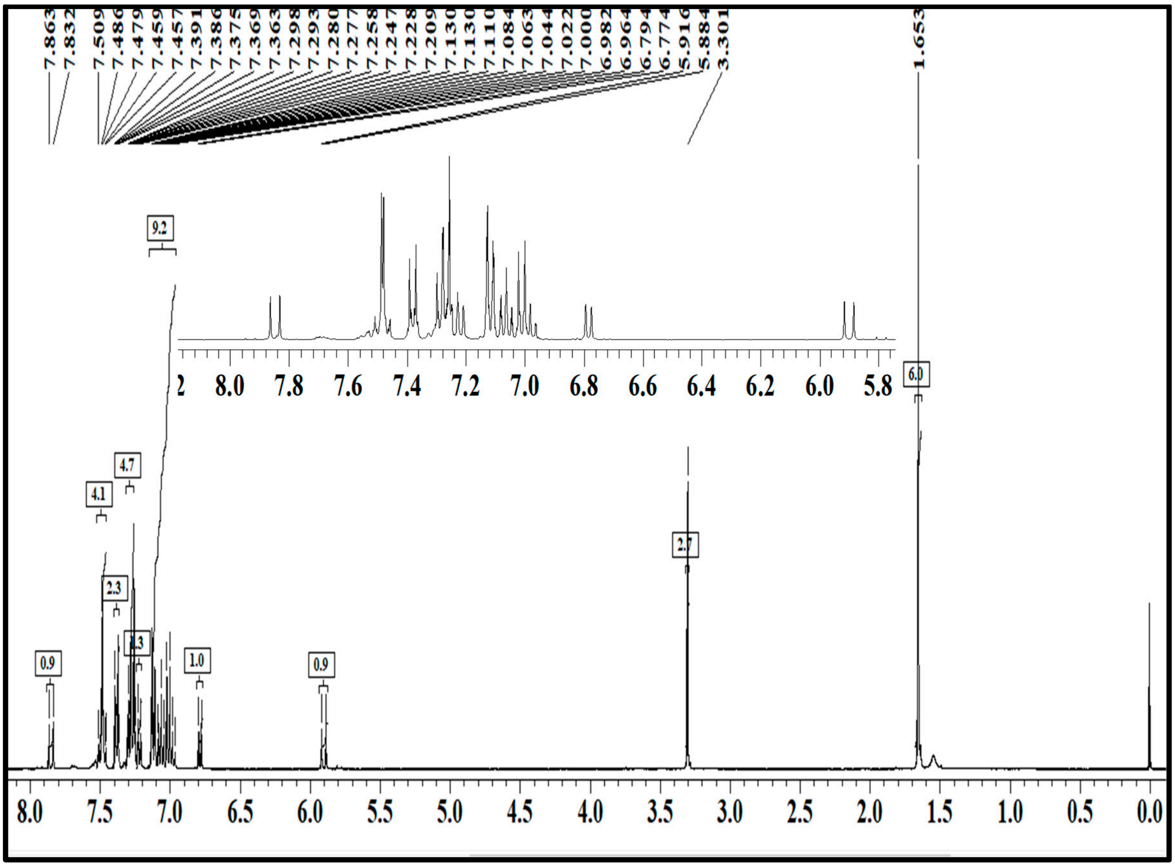


**Figure S16.** $^{1}$H NMR spectra of TPA-MC-1.

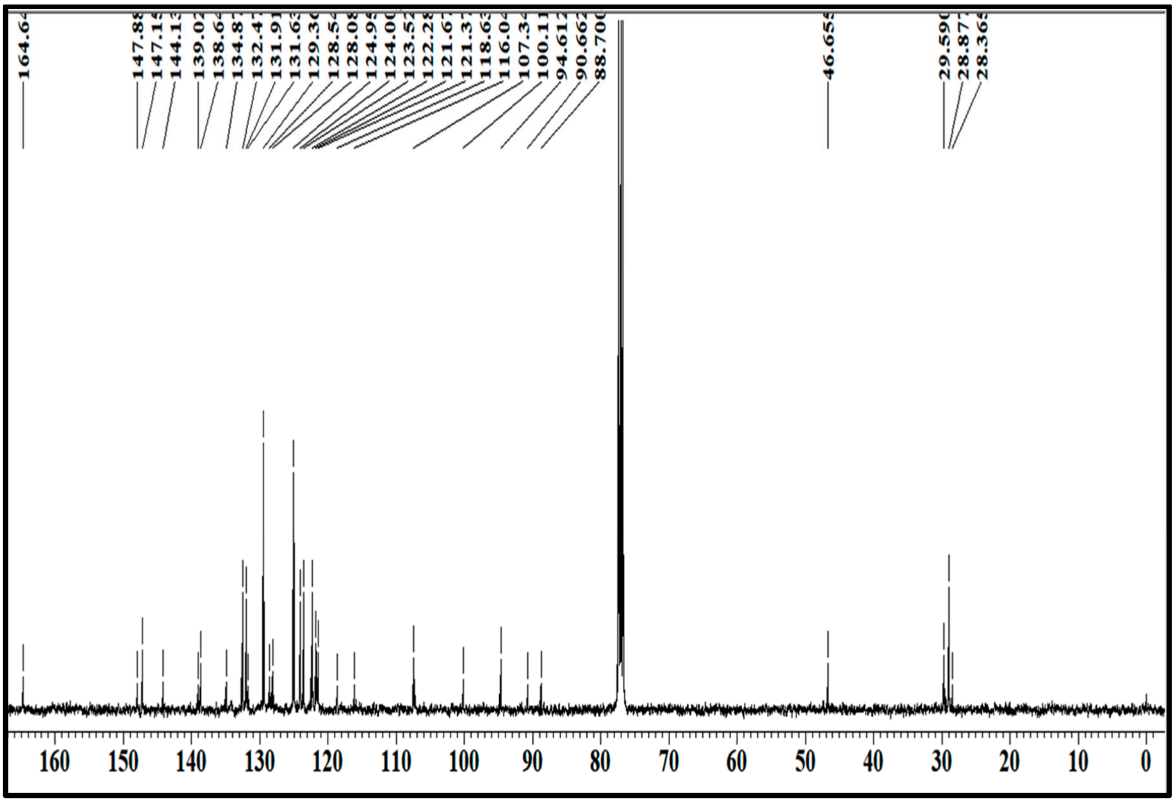


**Figure S17.** $^{13}$C NMR spectra of TPA-MC-1.

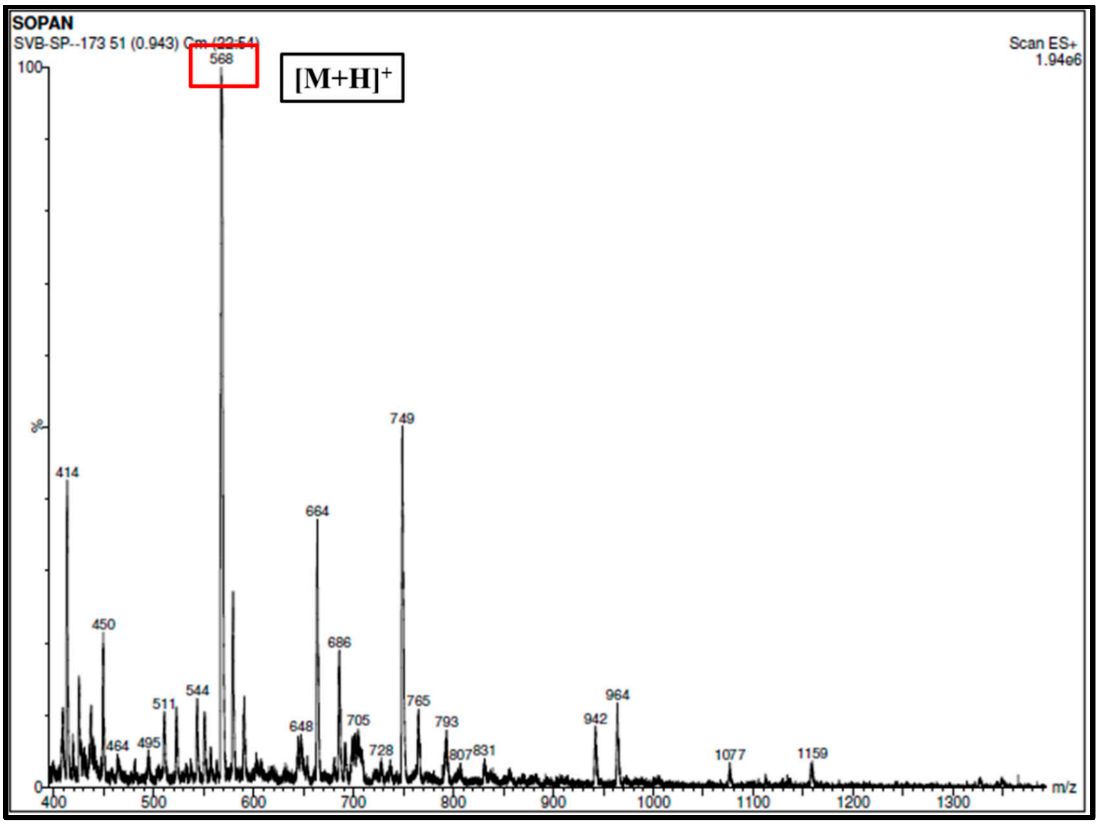


**Figure S18.** ESI-Mass of TPA-MC-1.

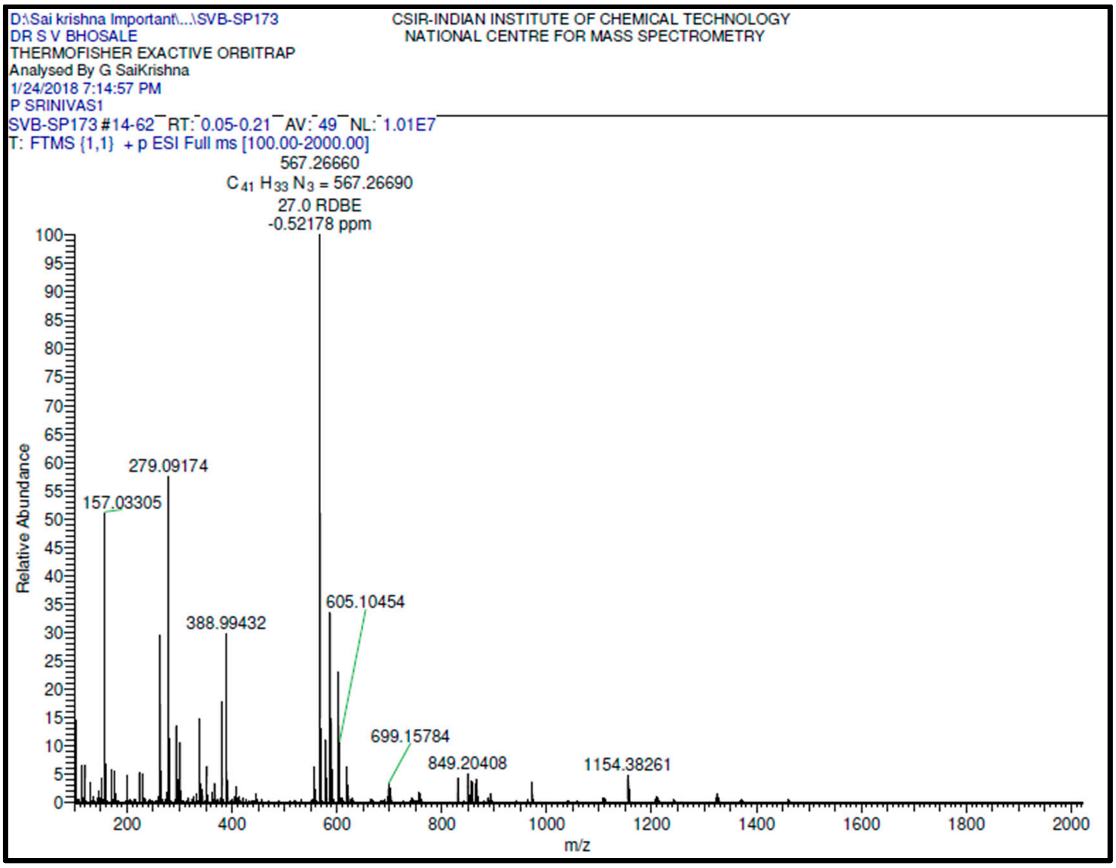


**Figure S19.** HRMS of TPA-MC-1.

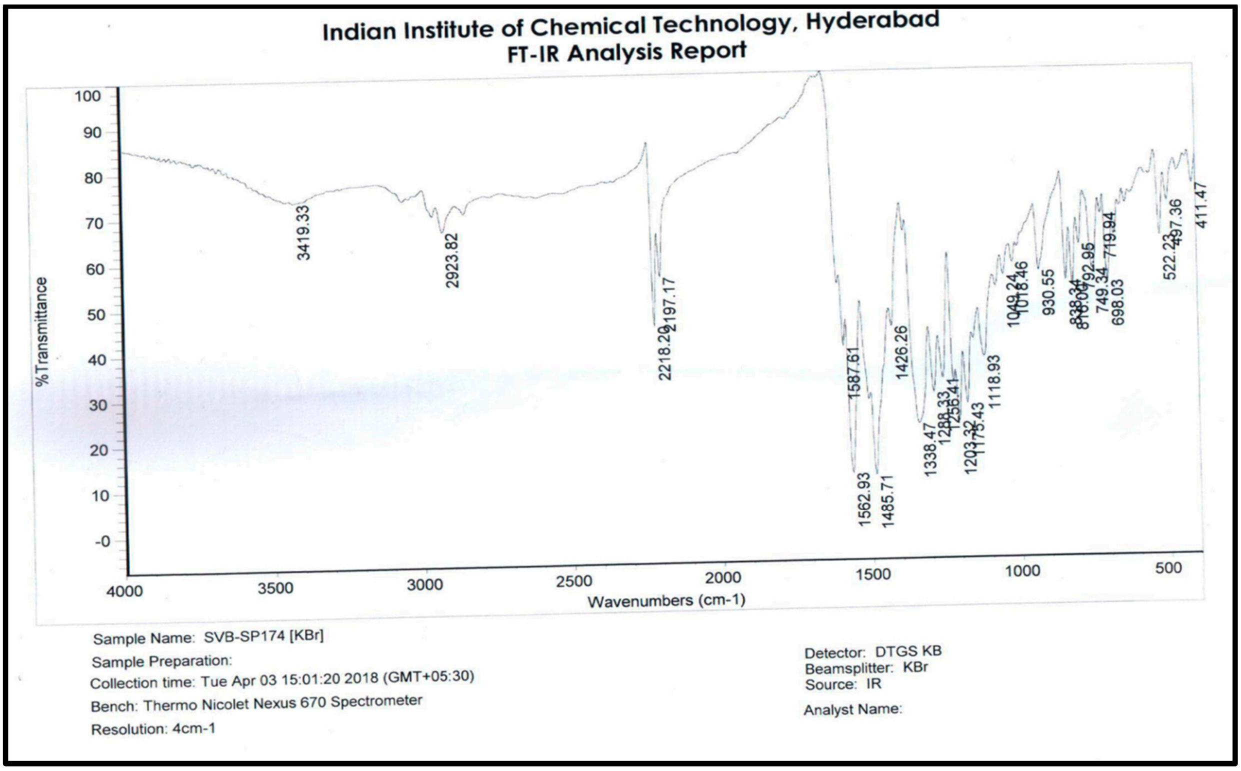


**Figure S20.** FT-IR spectra of compound TPA-MC-2.

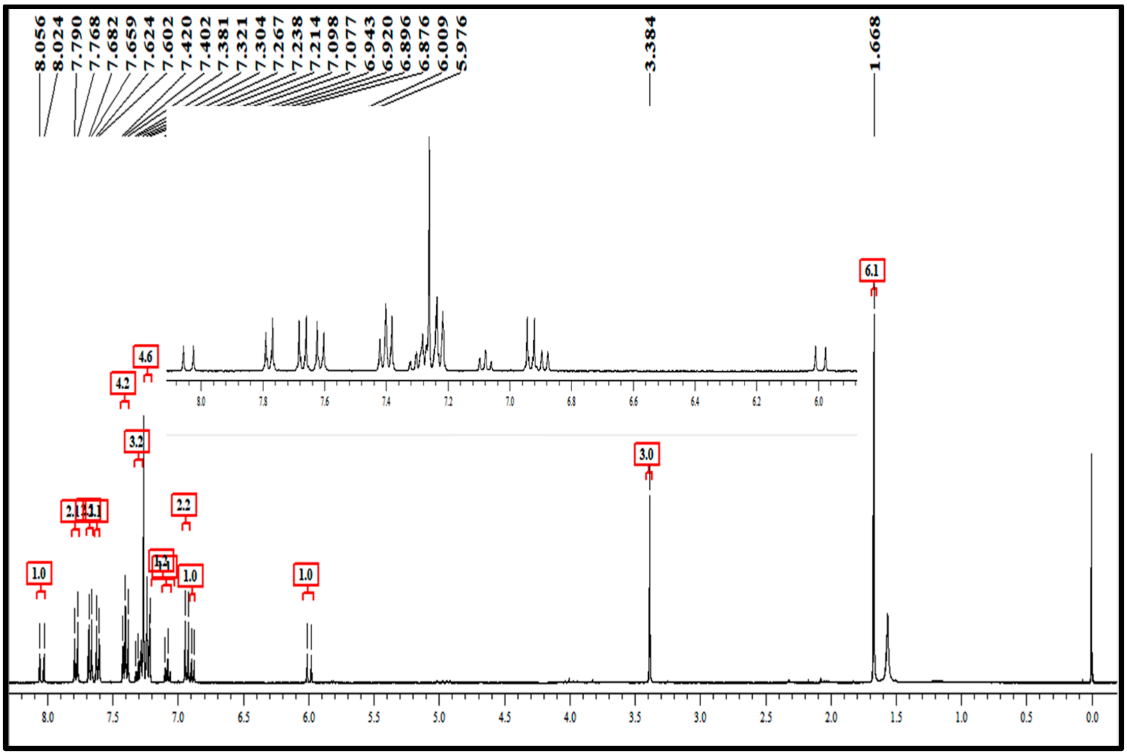

**Figure S21.** $^{1}H$ NMR spectra of TPA-MC-2.

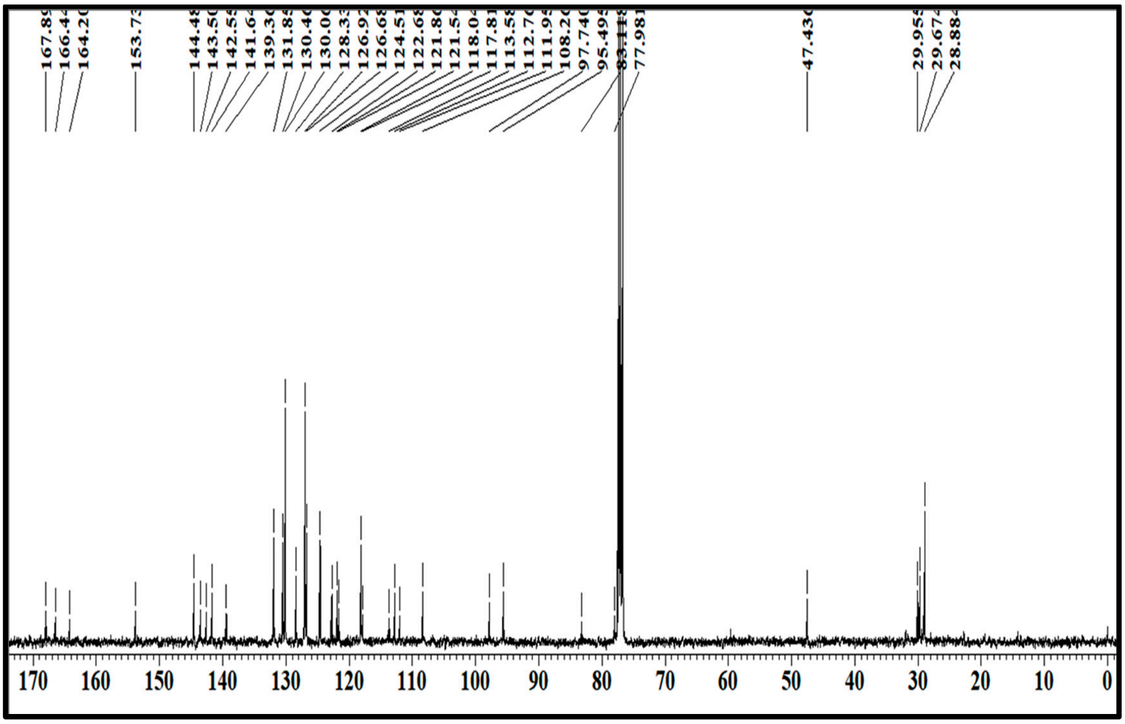

**Figure S22.** $^{13}C$ NMR spectra of TPA-MC-2.

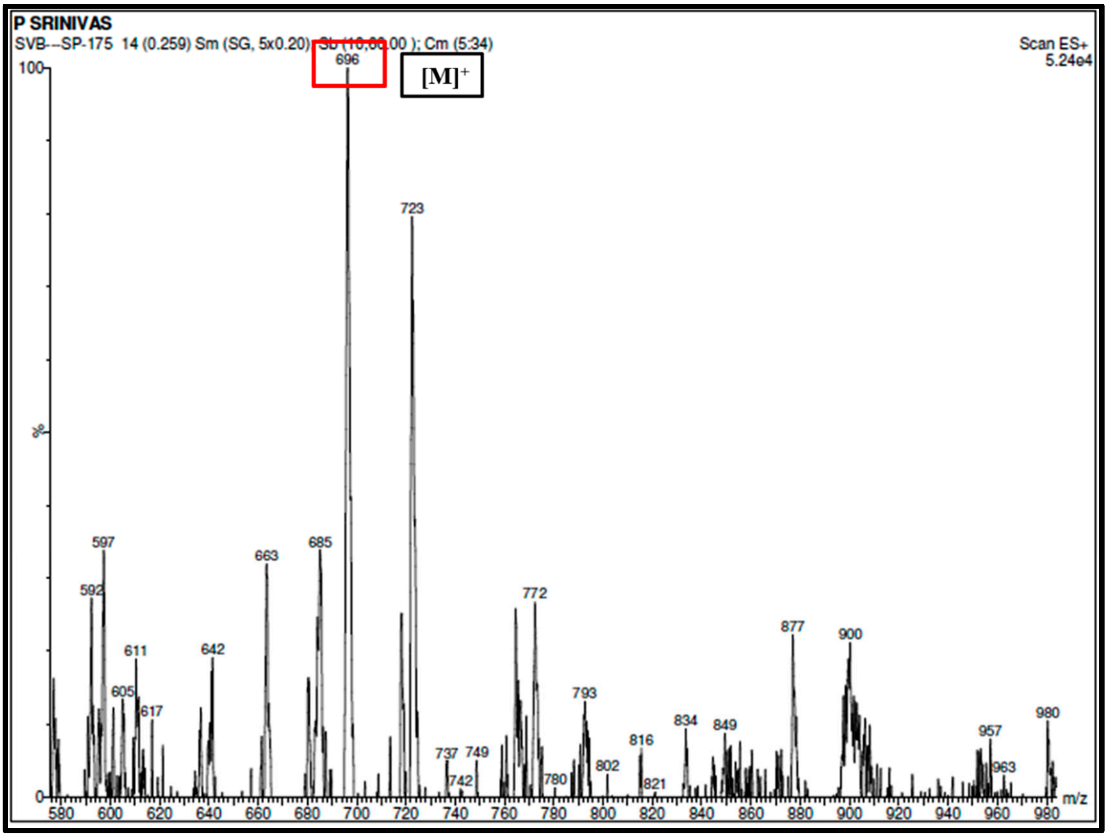


**Figure S23.** ESI-Mass of TPA-MC-2.

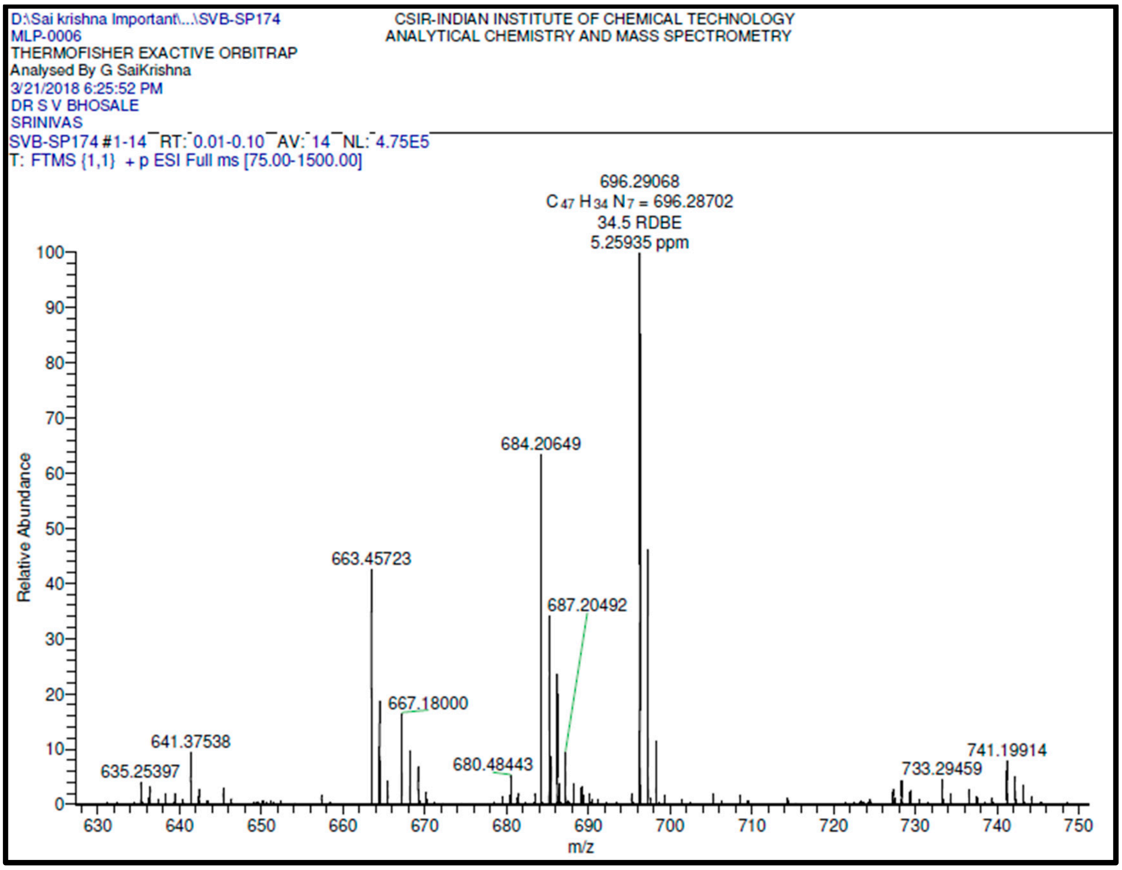


**Figure S24.** HRMS of TPA-MC-2.

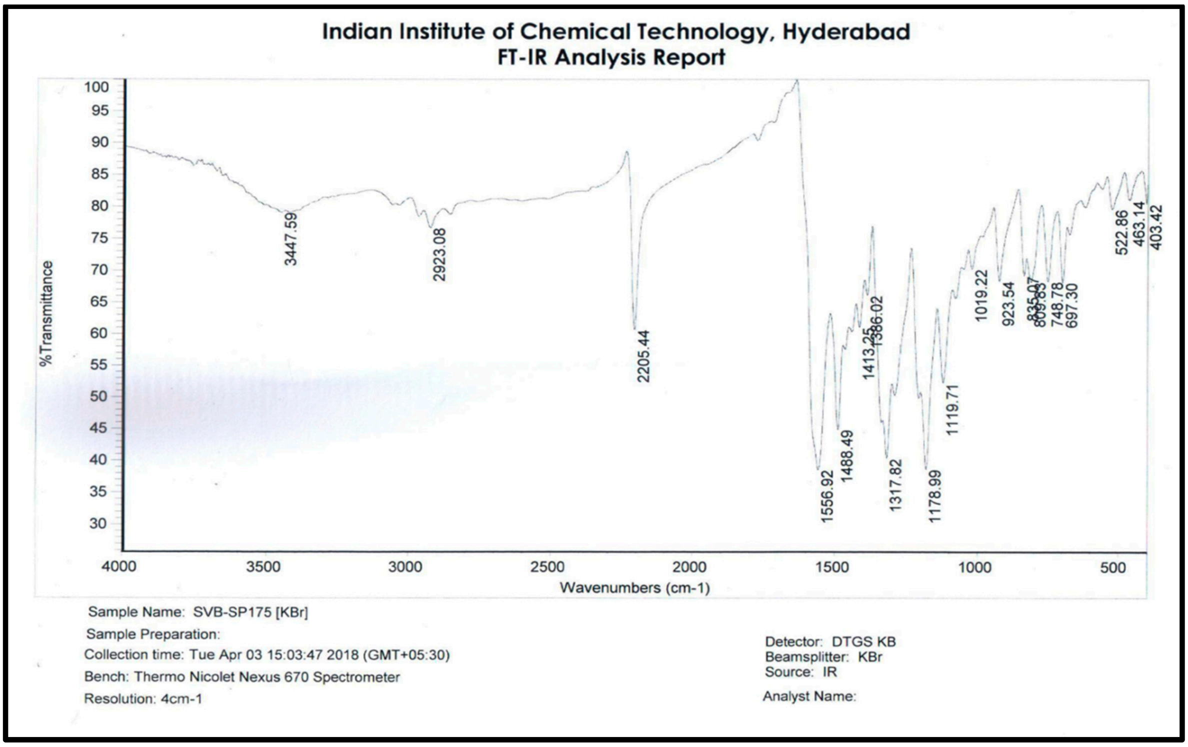


**Figure S25.** FT-IR spectra of compound TPA-MC-3.

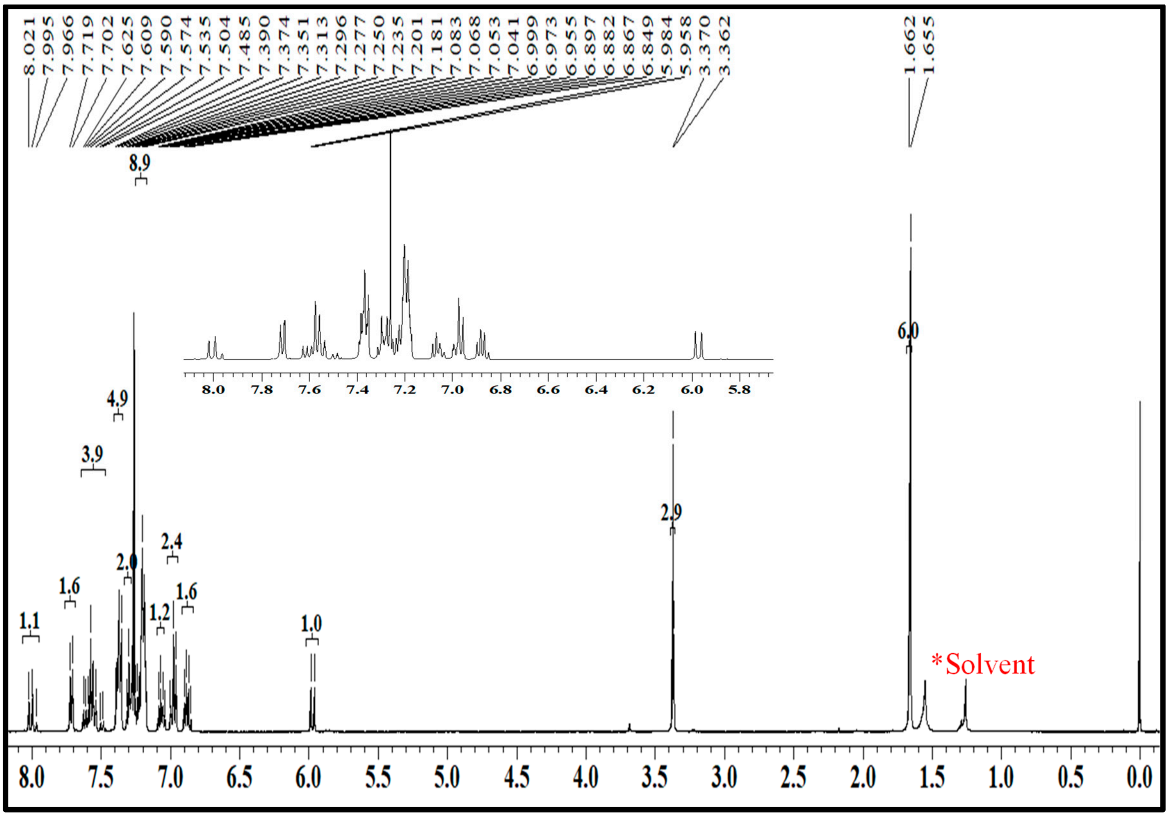


**Figure S26.** $^{1}$H NMR spectra of TPA-MC-3.

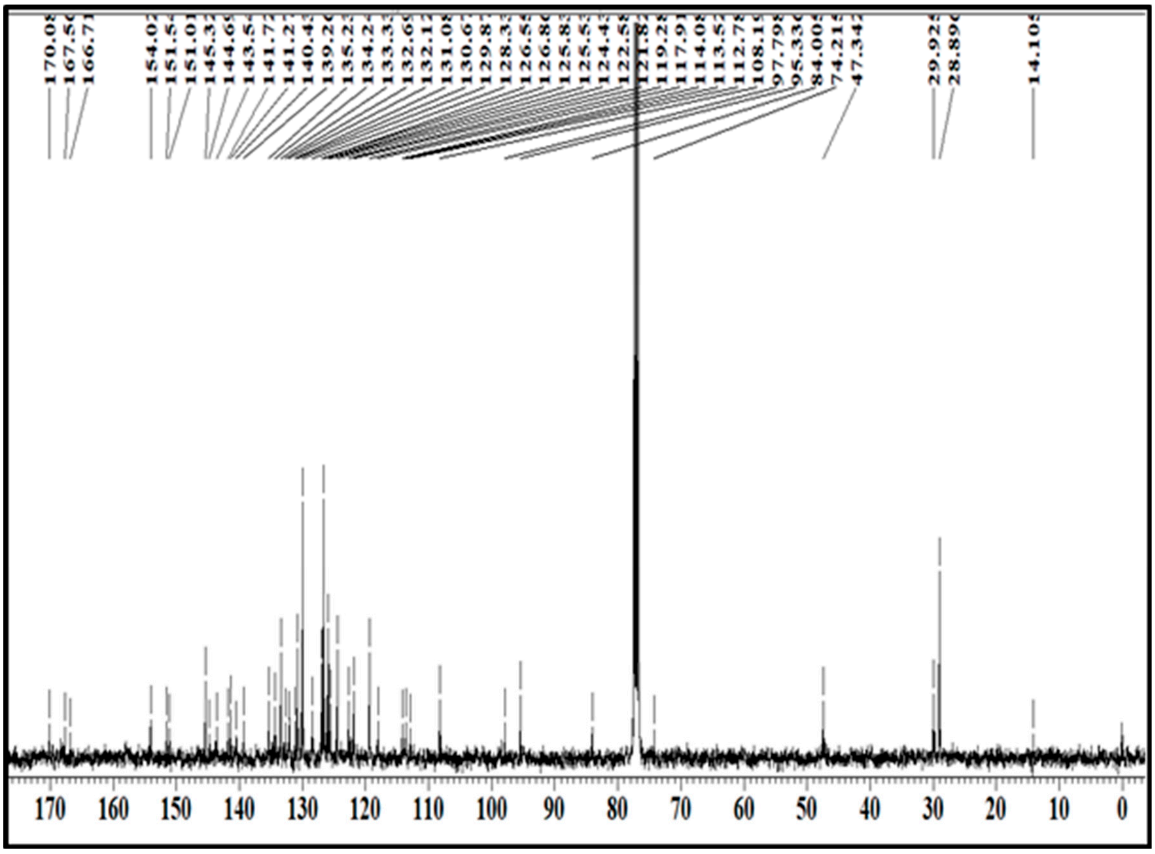


**Figure S27.** $^{13}C$ NMR spectra of TPA-MC-3.

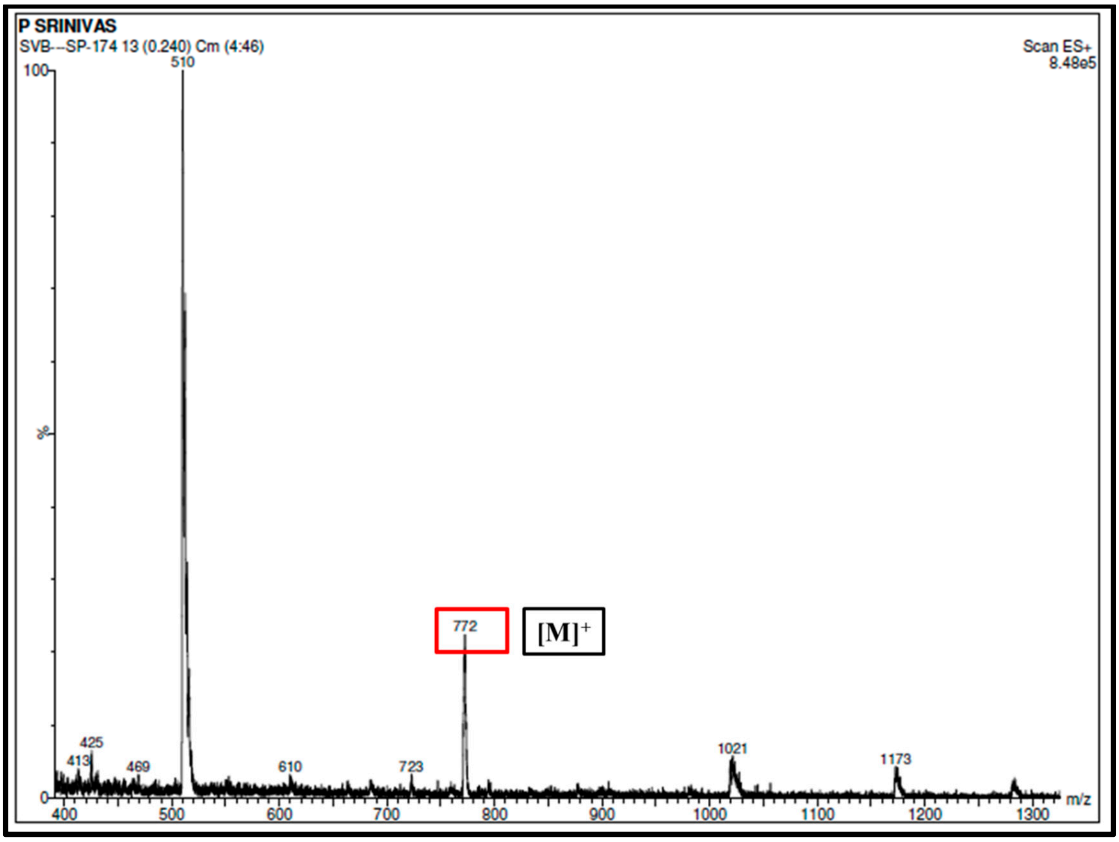


**Figure S28.** ESI-Mass of TPA-MC-3.

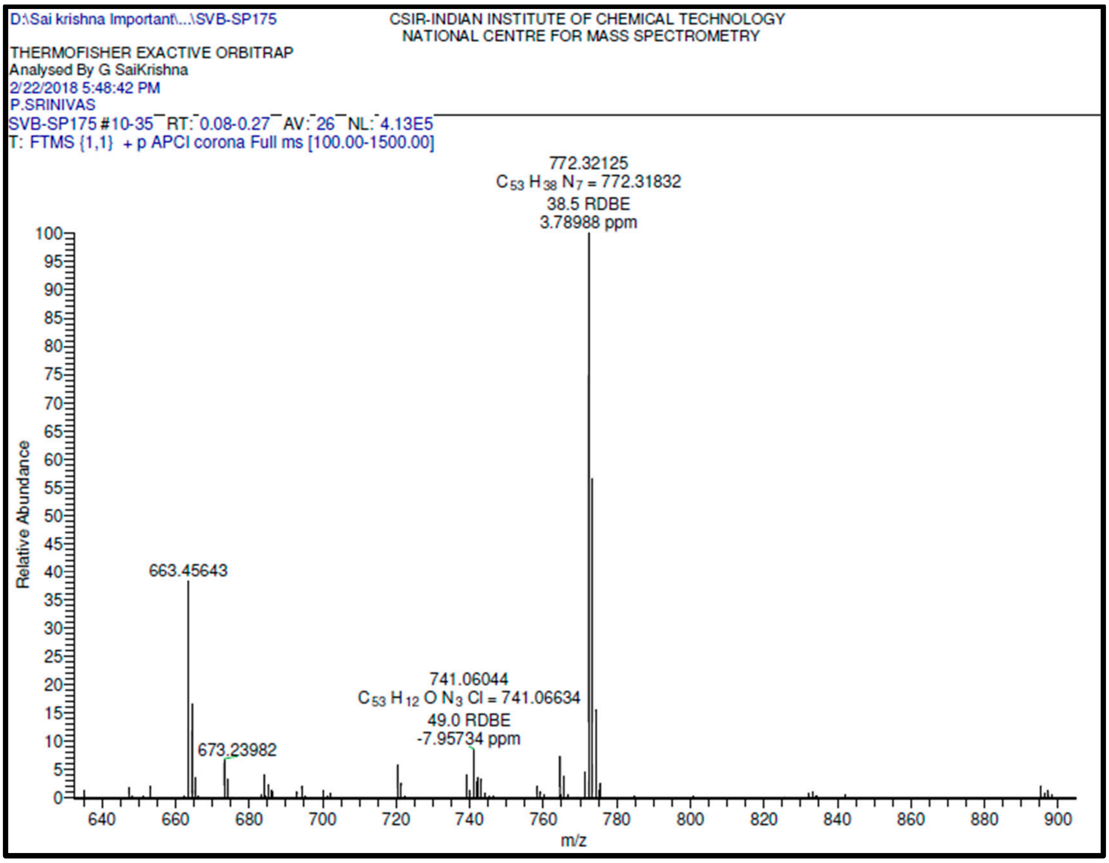


**Figure S29.** HRMS of TPA-MC-3.
